# Emotion Regulation and Aggression: A Systematic Review and Meta‐Analysis

**DOI:** 10.1002/ab.70055

**Published:** 2025-12-22

**Authors:** Kimberley Smith, Andrew Jones, Natasha Daly, Helena Widdrington, Carlo Garofalo, Steven M. Gillespie

**Affiliations:** ^1^ Department of Primary Care and Mental Health University of Liverpool Liverpool UK; ^2^ School of Psychology Liverpool John Moores University Liverpool UK; ^3^ Department of Philosophy, Social Sciences, and Education University of Perugia Perugia Italy

**Keywords:** emotion dysregulation, intimate partner, reappraisal, rumination, suppression, violence

## Abstract

Research on emotion regulation (ER) and aggression has increased rapidly in the past years, but heterogeneity across studies make integration of findings challenging. To estimate the size and consistency of the relationship between ER and aggression we conducted a systematic review and meta‐analysis. In total, 16,369 articles were retrieved from Scopus, PubMed, PsycINFO, and CINAHL, and 137 articles (171 studies) provided 918 effect sizes from a total sample of *N* = 252,605. Multilevel models showed significant pooled relationships, with small‐to‐moderate effects. Use of adaptive strategies was associated with lower aggression (*r* = −0.090, *I* ^2^ = 78%), but the precision estimate test (PET) bias corrected effect was non‐significant. Use of maladaptive strategies (*r* = 0.329, *I* ^2^ = 93%), and difficulties regulating emotions (*r* = 0.248, *I* ^2^ = 89%), were associated with higher aggression. *P‐*curves suggested little evidence of selective reporting while cumulative meta‐analysis showed relatively consistent effects over time. Moderator analyses showed that effects were generally stronger for physical and other forms of aggression than sexual aggression, and weaker for intimate partner targets than other targets. Difficulties in ER were most strongly related to reactive compared with proactive aggression, but the use of ER strategies did not differ between motivations. Our findings suggest that interventions targeting ER, including those that explore and challenge angry predispositions and provide skills to manage impulse control, may help to prevent aggressive behaviour.

Aggression refers to any behaviour, directed toward another, where the aggressor intends to cause proximate harm to the target, who aims to avoid it (Anderson and Bushman 2002 DeWall et al. 2011). Where there is intentional and severe physical harm to the target, these extreme acts of aggression are often referred to as violence. Recent figures show that an estimated 1 million violent offences, including cases of domestic abuse and intimate partner violence (IPV), ranging from minor assaults to murder, were recorded in the UK in the year ending December 2023 (Office of National Statistics 2024). Interpersonal violence has been recognized as an international public health concern (Krug et al. 2002), with acts of aggression having a lasting and negative impact on victims and their families (Krug et al. 2002). People who have a history of perpetrating aggression also tend to experience worse mental health outcomes, experience interpersonal and substance misuse difficulties, and are often involved in other criminal behaviours (Fazel et al. 2018 Farrington et al. 2017). Broader consequences for society include financial costs to the health service and the criminal justice system, and loss of workdays and productivity (Wolf et al. 2014 Senior et al. 2020;). A better understanding of the causes and correlates of aggression is therefore important for the development of rehabilitative interventions that support the prevention of aggression and violence.

## 1 Aggression

Two of the most influential models in aggression research are Buss and Perry (1992) classification, with physical (e.g., hitting, biting) and verbal (e.g., screaming, name calling) aggression representing the behavioural components of aggression, and anger and hostility representing affective and cognitive components, respectively, and Dodge and Coie (1987) model of reactive and proactive aggression. Reactive aggression is often perpetrated in response to a perceived threat or provocation and often accompanied by heighted physiological and emotional arousal, while proactive aggression is defined as aggressive acts with the motivation to cause harm and achieve personal gain (Raine et al. 2006). Recent research suggests that although dichotomic distinctions between reactive and proactive aggression are probably too rigid (Allen et al. 2018; Howard et al. 2009), both forms of aggression can be characterized by dysfunctional emotion regulation (ER) processes (Garofalo et al. 2020 Howard et al. 2009).

Other theoretical models have attempted to explain the causes of aggressive behaviour. For example, the *General Aggression Model* (GAM Allen et al. 2018) is a comprehensive, integrative framework, that considers the role of social, cognitive, personality, developmental, and biological factors, and brings attention to the impact of ER processes in the inhibition or perpetration of aggressive behaviour (Allen et al. 2018). Alternatively, the *I* ^*3*^ *Model* (pronounced ‘I‐cubed model’) (Finkel and Hall 2018) is a general‐purpose metatheory, providing a comprehensive framework for understanding the dynamic and multifaceted nature of aggression. According to the I^3^ Model, the likelihood and intensity of aggression is influenced by three orthogonal processes, referring to immediate environmental stimuli that might usually afford a proclivity to aggress, situational or dispositional factors that can influence this proclivity, and inhibiting influences that suppress or prevent aggressive behaviour, such as the capacity for ER.

Although many of these models emphasize the role of anger and hostility, other emotional experiences, both positive and negative, have also been linked with aggression. For example, negative emotions including frustration (Blair 2010), and shame (Elison et al. 2014 Velotti et al. 2017) have all been linked with heightened use of aggression, while Chester (2017) and Moore et al. (2024) have emphasized the hedonically pleasant and reinforcing qualities of aggression.

## 2 Emotion Regulation

One factor that is common to most theories of aggression is the role of ER processes, which play a key role in regulating angry and other emotional states (Allen et al. 2018; Finkel and Hall 2018). ER is a complex, multidimensional construct best defined as one′s ability to understand, influence and express emotions in response to situations or events (Gross 1998b) Gratz and Roemer (2004). propose that ER represents a collection of skills and abilities, including emotional awareness, clarity, and impulse control, that allow people to respond to emotion eliciting situations and events in ways that are aligned with their values, morals, and goals. The relative absence of any or all of these skills and abilities is indicative of difficulties in ER.

In contrast, the Process Model of ER (Gross 1998a, 2002; Gross and John 2003), suggests that people use different strategies to regulate their emotions at different points of the emotion generation process. These strategies are broadly divided into antecedent‐focused and response‐focused strategies. Antecedent‐focused strategies are typically used to change, reframe or reinterpret the emotion provoking situation before generating an emotional response, with example strategies including situation selection, situation modification, and cognitive change (e.g., attempts to reappraise or reframe a situation). Response‐focused strategies refer to efforts to directly modify an emotion after it has been generated and includes attempts to directly modify or suppress an emotional response. Although the Process Model distinguishes between antecedent and response focused strategies, it is also possible that antecedent focused strategies can continue to be used after an emotional response has been generated (Sheppes and Gross 2011). In this way, the process model views emotion generation as a dynamic process and the point in the process at which a particular strategy is used may influence its effectiveness and the intensity of the response to be regulated (Sheppes and Gross 2011). This model has widely been considered as a reference point for systematic reviews and meta‐analyses, including on the relationship of ER with suicidality (Rogier et al. 2024), psychopathology (Compas et al. 2017), and the effectiveness of different ER strategies (Webb et al. 2012).

The extent to which different strategies for ER are considered adaptive versus maladaptive has been contested, and it has been proposed that the outcome of each strategy is dependent on the situation and personal context rather than being inherently adaptive or maladaptive (Aldao et al. 2015). However, others have made distinctions between the outcomes associated with different strategies when applying them to anger and aggression, arguing that some strategies (e.g., suppression) are associated with more negative long‐term outcomes, including maintaining hostile thoughts and heightened angry arousal (Roberton et al. 2012 Davey et al. 2005).

## 3 The Current Review

Earlier systematic reviews have narratively synthesized the relationship of aggression with ER strategy use in young people and adults (Navas‐Casado et al. 2023), and with emotional intelligence (García‐Sancho et al. 2014), mindfulness (Gillions et al. 2019), and emotional and social information processing (Smeijers et al. 2020), but there has been no quantitative synthesis of the relationship of ER with aggression. Individual studies vary widely in the population being sampled, how both constructs are operationalized, and the strength of the association can differ depending on factors such as the type (physical, sexual, and other) and the target (intimate partner *vs.* other) of aggression, the motivation for aggression (e.g., reactive/impulsive *vs.* proactive/instrumental), and gender. In the current review, we aimed to not only quantify the strength and consistency of the relationship of aggression with adaptive and maladaptive strategy use, and difficulties in ER, but also to identify key moderators and examine methodological quality of the individual studies and potential publication bias.

## 4 Methods

### 4.1 Transparency and Openness

We followed the Preferred Reporting Items for Systematic review and Meta‐Analysis framework (Page et al. 2021). All data and code are available on the Open Science Framework (https://osf.io/t7wa6/). The review was preregistered on the International Prospective Register of Systematic Reviews (PROSPERO 2024; CRD42023444732) in August 2023.

### 4.2 Search Strategy

We systematically searched four major electronic databases: Scopus, PsycINFO, CINAHL, and PubMed, from their earliest records until October 30th, 2024, for relevant titles and abstracts. The search strategy included a combination of terms associated with ER (emotion regulation OR emotion dysregulation OR reappraisal OR distraction OR situation modification OR rumination OR affect regulation OR affect dysregulation OR mood regulation OR mood dysregulation OR appraisal OR suppression OR situation selection OR attention deployment OR response modulation or cognitive change) and aggression (aggression OR aggressive OR violent OR violence). The search term NOT (child*) was applied to further filter records.

### 4.3 Study Eligibility

Studies were included in the review if (1) all participants were aged at least 16 years and the sample had a mean age of 18 years or older, (2) participants were not selected for on the presence of an intellectual or developmental disability or disorder (IQ lower than 70, ASD or ADHD); (3) studies were peer‐reviewed articles including quantitative data; (4) written in English; and (5) employed validated measures of both aggression and ER. Participants could be from any setting, including samples from the community or clinical and/or forensic populations. For offender samples, acts of aggression could include sexual offences, but studies of people who had sexually offended exclusively online were excluded, given known differences between contact and online sexual offenders (Babchishin et al. 2011). Theses, case reports and reviews were excluded. Online forms of abuse including cyber aggression, and other forms of aggression such as driving aggression and displaced relational aggression, were excluded.

Subscales that measured anger rumination were included as measures of ER, even where the overall measure was intended as a measure of aggression. Measures of mindfulness, alexithymia, distress tolerance, impulsivity, and information processing were excluded. Some measures of emotional intelligence share considerable conceptual overlap with measures of ER. For example, the emotional intelligence subscale of the Trait‐Meta Mood Scale (TMMS Salovey et al. 1995) shares conceptual overlap with emotional awareness and emotional clarity, and partial overlap with ER strategies subscales of the Difficulties in Emotion Regulation Scale (DERS Gratz and Roemer 2004). Therefore, a decision was made to include this subscale of the TMMS, while excluding all other measures of emotional intelligence. Where measures of ER or aggression included subscales that did not meet the inclusion criteria, effect sizes for those subscales were excluded from the review (e.g., the Injury and Negotiation subscales of the Conflict Tactics Scale (Straus et al. 1996).

### 4.4 Study Selection

Studies were extracted and reviewed using a two‐step process, with screening and selection of titles and abstracts, followed by decisions based on full text review. KS and ND both independently reviewed the titles and abstracts of all papers, and all papers that proceeded to full‐text review. Any discrepancies were discussed and resolved between the two reviewers. Screening was completed using EndNote and Rayyan software.

### 4.5 Data Extraction

Data extracted from each paper included title, author/s, year of publication, location, number of participants, type of sample (e.g., college, community, clinical), age (mean, standard deviation or range), gender, ethnicity/nationality, aggression measures included, ER measures included, and the correlation coefficient values for the relationship between the ER and aggression scales and/or subscales (*r* value and statical significance) or vales (*M*, SD, *n)* to calculate a standardized mean difference (SMD) between e.g., high and low aggression groups. Continuous measures of the relationship between ER and aggression were prioritized where possible. All data were independently extracted by KS and ND with any discrepancies resolved between raters.

Where subscales of ER and aggression measures were available, we extracted data relevant to subscales rather than total scores for inclusion in multilevel meta‐analyses. Consistent with earlier reviews (Rogier et al. 2024), we categorized ER scales/subscales as measures of either adaptive (e.g., reappraisal) or maladaptive (e.g., suppression) strategies, or as competency or difficulty‐based measures (e.g., subscales of the DERS Gratz and Roemer 2004). Types of aggression were categorized as physical, sexual (i.e., aggressive acts with a sexual element), or other, including verbal and psychological aggression, anger, hostility, and controlling behaviours. Total scores that included the sum of different types of aggression were also coded as other. Finally, measures of aggression were coded based on whether the target of aggression was an intimate partner versus another/unknown target, and whether the motivation was proactive/premeditated versus reactive/impulsive.

### 4.6 Statistical Analysis

Multilevel meta‐analytic models were calculated to quantify the size and consistency of the relationship of aggression with adaptive and maladaptive strategy use, and competency or difficulties in ER. In accordance with recommendations (Polanin and Snilstveit 2016), we converted all effect sizes to a common effect size (*r*). Where effect sizes were unavailable for individual scales or subscales, we used the total score. We used the ‘effectsize’ package (Ben‐Shachar et al. 2020), in *R* (R Core Team 2018), to calculate and convert between effect sizes. SMD effect sizes were calculated for studies of between subject designs using the ‘escalc’ function, before using the ‘d_to_r’ function to convert between effect sizes. Fisher′s *Z* transformation was used to improve the distribution of the coefficients, and findings were back‐transformed for presentation in text and orchard plots. Common language effect sizes were also used to aid interpretation (Mastrich and Hernandez 2021). For correlations, the formula for the common language effect is

CLr=(arcsin(r)/π)+.5

and the value is the probability of any individual with a higher score for aggression also having a higher score for ER.

As several studies included more than one eligible effect size, we conducted multilevel, random‐effects meta‐analyses, in which effect sizes were nested within studies, using the ‘rma.mv’ function of the ‘metafor’ package (Viechtbauer 2010), in *R* (R Core Team 2018). *I* ^*2*^ was used as the measure of heterogeneity, with values > 50% indicative of moderate heterogeneity, and > 75% indicative of substantial heterogeneity. We also performed a series of moderator analyses on the type (physical *vs.* sexual *vs.* other), target (IPV *vs.* other), and motivation (proactive/premeditated *vs.* reactive/impulsive) for aggression, and gender. Finally, we conducted a meta‐analysis including only effect sizes generated from subscales of the DERS (Gratz and Roemer 2004) and conducted a moderator analysis to understand differences in the size of the relationship of DERS subscales with aggression.

### 4.7 Publication Bias

Typical methods for the assessment of publication bias, including Egger's test and Trim and Fill analyses, are not optimized for multilevel models. We attempted to overcome this issue by running these on single level models. We also report funnel plots for each pooled model (Mavridis and Salanti 2014) and include precision‐effect testing and precision‐effect estimate with standard error (PET‐PEESE) analyses. PET/PEESE analyses regress observed effect sizes on their standard errors and squared standard errors, respectively. A significant slope in the PET model indicates the presence of small‐study (publication) bias (e.g., the effect size is associated with precision), while the intercepts from the PET‐PEESE models provide bias‐corrected estimates of the true underlying effect size. Cumulative meta‐analyses based on year of publication and *p*‐curve analyses were also conducted and reported in full in Supplemental Material.

### 4.8 Quality Assessment

The Critical Appraisal Tool for Cross‐Sectional Studies (AXIS Downes et al. 2016) was used to assess the methodological quality and risk of bias for all papers included in the review. The tool provides a 20‐item checklist allowing the rater to undertake a complete assessment of the methodological quality of each component of the study, including the reporting of aims, design, methodology, results, and discussion. All full‐texts were quality assessed by KS, and 10% of full‐texts were independently quality assessed by HW. No major discrepancies emerged, and any disagreements were discussed to achieve a consensus rating.

## 5 Results

### 5.1 Study Selection

**Figure 1:**
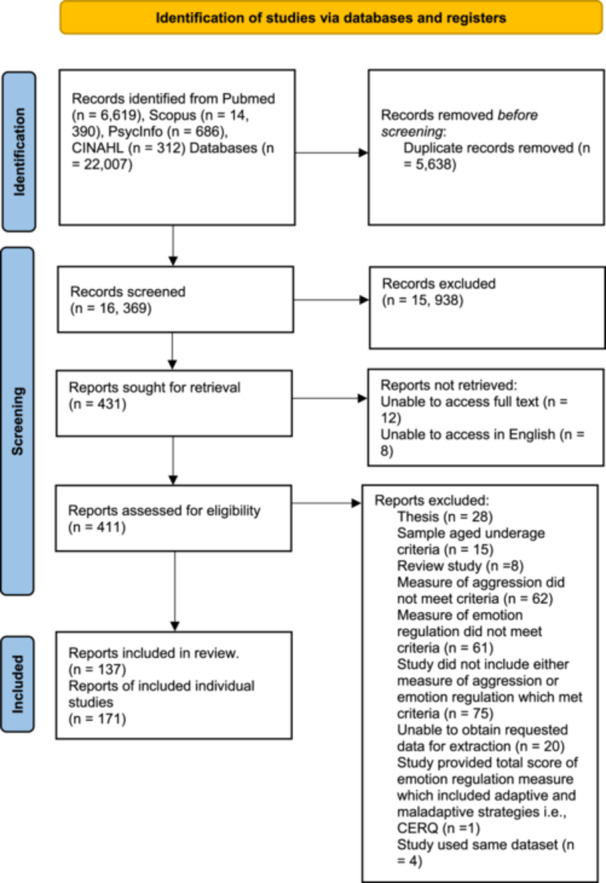
PRISMA flow diagram summarizing the screening process for eligible articles. [Color figure can be viewed at wileyonlinelibrary.com]

A total of 22,007 papers were identified. Following the removal of duplicates, the titles and abstracts of 16,369 individual papers were screened. Of these, 431 papers were retained for full text review, with 157 papers considered eligible for inclusion. Relevant effect sizes were available for 128 papers. Authors of the remaining 29 papers were contacted via email to request the relevant effect sizes, or the raw data, and nine responses were received. In total, 137 papers with available effect sizes of interest were included in meta‐analyses. Thirty‐one papers reported on more than one study (1/31 included four studies), representing a total of 171 studies overall. Figure 1 outlines the full selection process, using the PRISMA 2020 flow diagram for new systematic reviews including searches of databases, registers, and other sources (Page et al. 2021).

### 5.2 Sample Characteristics

Sample characteristics are displayed in Table S1 in Supplemental Material. Seventy‐six studies recruited samples of undergraduate/college participants, 58 included a community sample, and 18 studies included clinical samples of psychiatric inpatients or people with diagnoses of personality disorder, schizophrenia and bipolar disorder, substance misuse, gaming disorder, or participants recruited from interventions or community/therapy programs. Nineteen studies included offender samples. Sample sizes ranged from 13 to 1845, with an overall sample size of *N* = 252,605. The age of participants ranged from 16 to 96 years. Most studies included samples with male and female participants (94/171), 54 studies were restricted to only male participants, and 23 included only female participants. Of the studies that reported on ethnicity (76/171), participants in 63 studies were predominantly White (proportion ranging from 42% to 100%).

### 5.3 Study Characteristics

Study characteristics are displayed in Table S1 in Supplemental Material. Included studies were conducted around the globe, including in North America (*n* = 74), Italy (*n* = 24), and China (*n* = 22). Most studies were cross‐sectional (155/171), while six studies were longitudinal. Publication dates ranged from 2007 to 2024, with 85% of studies being published after 2015.

Tables S2 and S3 in Supplemental Material show a full list of all ER measures and aggression measures, respectively, and their coding for moderator analyses. One hundred studies included ER measures that were categorized as assessing competency or difficulties in ER, with 92 studies using the DERS or its subscales (Gratz and Roemer 2004). Seventy‐seven studies included effect sizes related to the use of maladaptive strategies, including rumination, hostile rumination, anger rumination, suppression, and avoidance. Twenty studies included effect sizes related to the use of adaptive strategies, including reappraisal, acceptance, emotional concealment/adjustment/tolerance, positive refocusing, and refocus on planning and putting into perspective.

Sixty‐five studies used the Buss–Perry Aggression Questionnaire (BPAQ Buss and Perry 1992) and its short form (2/65) to measure aggression. Forty‐two studies used versions of the Conflict Tactics Scale‐2 or its subscales (CTS2 Straus et al. 1996) to measure aggression towards an intimate partner, including physical aggression, psychological aggression, and sexual aggression.

### 5.4 Quantitative Synthesis

#### 5.4.1 Adaptive Strategies

**Figure 2:**
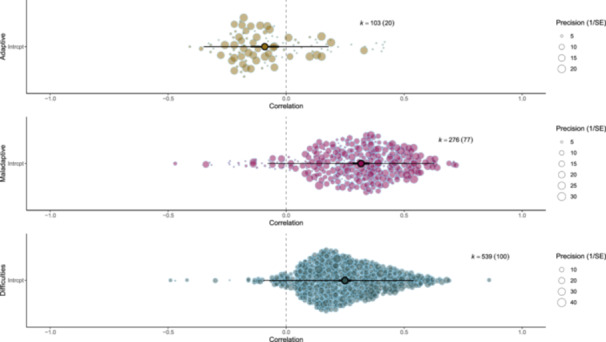
Orchard plot showing the meta‐analytic mean and individual effect sizes scaled by their precision for the relationship of adaptive and maladaptive ER strategies, and difficulties in ER, with aggression. [Color figure can be viewed at wileyonlinelibrary.com]

**Figure 3:**
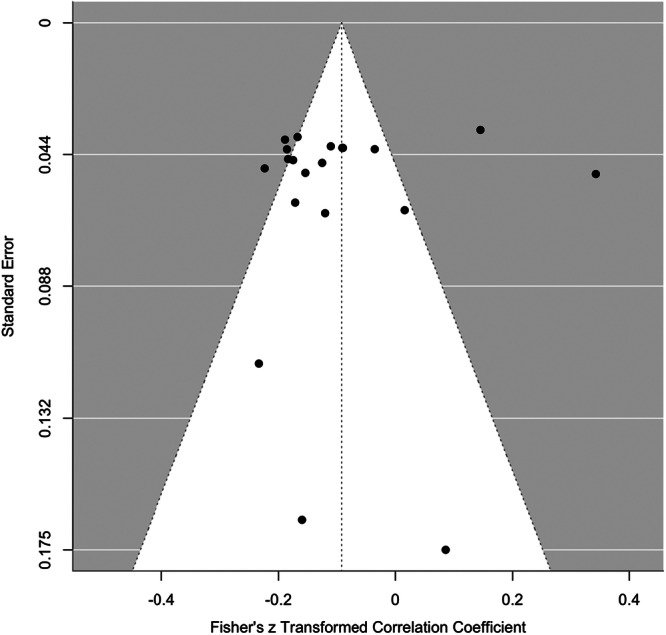
Funnel plot showing publication bias for the association between adaptive ER strategies and aggression.

A multilevel model fit the data better than a single level model (χ^2^(1) = 44.62, *p* < 0.001). An orchard plot in Figure 2 shows that there was a small negative pooled association between use of adaptive ER strategies and aggression across 103 effect sizes from 20 studies (*r* = −0.090 [95% CI: −0.030 to −0.150], *p* < 0.001, *I* ^*2*^ = 78%), with most of the variance ( ~ 76%) attributable to differences between studies. The common language effect size statistic suggests that for any two people selected at random, a person with a higher value of aggression will have a lower value on adaptive strategies 52.7% of the time. Egger's test was significant (*z* = 2.11, *p* = 0.035), indicating evidence of publication bias. Trim and Fill models suggested the imputation of five effect sizes in a single level model to improve the symmetry of the funnel plot (see Figure 3), which had a negligible influence on the overall pooled effect (*r* = −0.085 [95% CI: −0.052 to −0.122]). The PET slope was not significant, indicating no evidence of small‐study or publication bias. The PET intercept, which provides the bias‐corrected effect, was also not statistically significant, (*r* = −0.106, 95% CI [–0.210, 0.002]). This suggests that, after adjusting for potential bias, the estimated effect is small, negative, and not significant. Cumulative meta‐analyses by year of publication demonstrated consistent effects over time (see Figure S1, Supplemental Material). Greater distribution of *p*‐values closer to 0.01 compared to 0.05 suggests limited evidence of selective reporting of effects (see Figure S2, Supplemental Material).

Type of aggression significantly moderated the relationship between adaptive strategies and aggression (χ^2^(2) = 6.45, *p* = 0.039). The association for physical aggression (*n* = 23 effects from 7 studies: *r* = −0.135 [95% CI: −0.062 to −0.207]; *I* ^*2*^ = 41%) was significantly different (*p* = 0.011) to sexual (*n* = 18 effects from 2 studies: *r* = 0.041 [95% CI: −0.361 to 0.430]; *I* ^*2*^ = 67%) but did not differ (*p* = 0.891) from other types of aggression (*n* = 62 effects from 20 studies: *r* = −0.095 [95% CI: −0.036 to −0.153]; *I* ^*2*^ = 81%). There was also a significant difference in the strength of association for sexual aggression versus other types of aggression (*p* = 0.022). Note, that all the effect sizes for use of adaptive strategies with sexual aggression were taken from one paper providing two studies. The target of aggression did not significantly moderate the association (χ^2^(1) = 1.42, *p* = 0.231), with the strength of association similar for an intimate partner (*n* = 68 effects from 6 studies: *r* = −0.053 [95% CI: 0.060 to −0.164]; *I* ^*2*^ = 59%) versus other targets (*n* = 35 effects from 16 studies: *r* = −0.118 [95% CI: −0.057 to −0.177]; *I* ^*2*^ = 86%). Only two studies (four effects) measured the association of adaptive strategies with reactive and proactive aggression, meaning the moderator effect could not be tested. Gender did not significantly moderate the strength of the association (χ^2^(1) = 0.46 *p* = 0.494), with the strength of association similar for males (*n* = 67 effects from 6 studies: *r* = −0.107 [95% CI: −0.020 to −0.191]; *I* ^*2*^ = 40%) and females (*r* = −0.185 [95% CI: −0.132 to −0.232]; *I* ^*2*^ = 0%). Of note, all effect sizes for females included in the moderator analysis were taken from one study.

#### 5.4.2 Maladaptive Strategies

**Figure 4:**
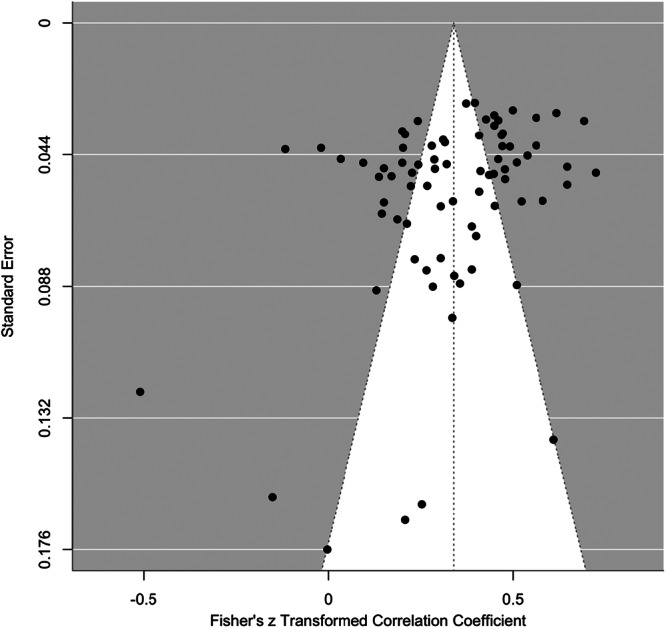
Funnel plot showing publication bias for the association between maladaptive ER strategies and aggression.

A multilevel model fit the data better than a single level model (χ^2^ (1) = 53.25, *p* < 0.001). An orchard plot in Figure 2 shows that there was a significant positive pooled association between use of maladaptive ER strategies and aggression across 276 effect sizes from 77 studies (*r* = 0.329 [95% CI: 0.282–0.354], *p* < 0.001; *I* ^*2*^ = 93%), with most of the variance ( ~ 54%) attributable to effect sizes within studies. The common language effect size suggests that for any two people selected at random, a person with a higher value of aggression will have a higher value on maladaptive strategies 61% of the time. Egger's test was significant (*z* = 6.78, *p* < 0.001), indicating evidence of publication bias. Similarly, Trim and Fill models suggested the imputation of 60 effect sizes in a single level model to improve the symmetry of the funnel plot (see Figure 4), which had a small influence on the overall pooled effect (*r* = 0.388 [95% CI: 0.359–0.417]). The PET slope was significant (*p* < 001), indicating evidence of small study effects. The bias corrected PEESE model showed a significant positive association (*r* = 0.355 [95% CI: 0.318–0.393]), indicating that even after adjusting for potential publication bias, a moderately sized positive effect remained. Cumulative meta‐analyses by year of publication demonstrated consistent effects over time (see Figure S1, Supporting Information). Greater distribution of *p*‐values closer to 0.01 compared to 0.05 suggests limited evidence of selective reporting of effects (see Figure S3, Supporting Information).

Type of aggression significantly moderated the relationship between maladaptive strategies and aggression (χ^2^ (2) = 9.65, *p* = 0.008). The association for physical aggression (*n* = 62 effects from 29 studies: *r* = 0.247 [95% CI: 0.191–0.301]; *I* ^*2*^ = 89%) was not significantly different (*p* = 0.265) to sexual aggression (*n* = 16 effects from 3 studies: *r* = 0.222 [95% CI: 0.103–0.334]; *I* ^*2*^ = 40%), but did significantly differ (*p* = 0.012) from other types of aggression (*n* = 198 effects from 69 studies: *r* = 0.334 [95% CI: 0.294–0.373]; *I* ^*2*^ = 94%). There was also a significant difference in the strength of association for sexual aggression versus other types of aggression (*p* = 0.022). The target of aggression also significantly moderated the relationship (χ^2^ (1) = 8.43, *p* = 0.003), with a weaker association for intimate partner targets (*n* = 66 effects from 13 studies: *r* = 0.203 [95% CI: 0.120–0.284]; *I* ^*2*^ = 67%), versus other (*n* = 210 effects from 67 studies: *r* = 0.337 [95% CI: 0.301–0.372]; *I* ^*2*^ = 94%). Motivation for aggression did not significantly moderate the relationship (χ^2^ (1) = 2.11, *p* = 0.146). The association for proactive aggression (*n* = 13 effect sizes from 12 studies: *r* = 0.330 [95% CI: 0.190–0.456]; *I* ^*2*^ = 97%) was not significantly different to reactive aggression (*n* = 14 effects from 13 studies: *r* = 0.437 [95% CI: 0.334–0.530]; *I* ^*2*^ = 96%). Gender did not moderate the strength of the association (χ^2^ (1) = 4.86, *p* = 0.027), with the association being significantly similar for males (*n* = 76 effects from 16 studies: *r* = 0.209 [95% CI: 0.129–0.284]; *I* ^*2*^ = 62%) than females (*n* = 5 effects from 4 studies: *r* = 0.266 [95% CI: 0.164–0.362]; *I* ^*2*^ = 59%).

#### 5.4.3 Difficulties in Emotion Regulation

**Figure 5:**
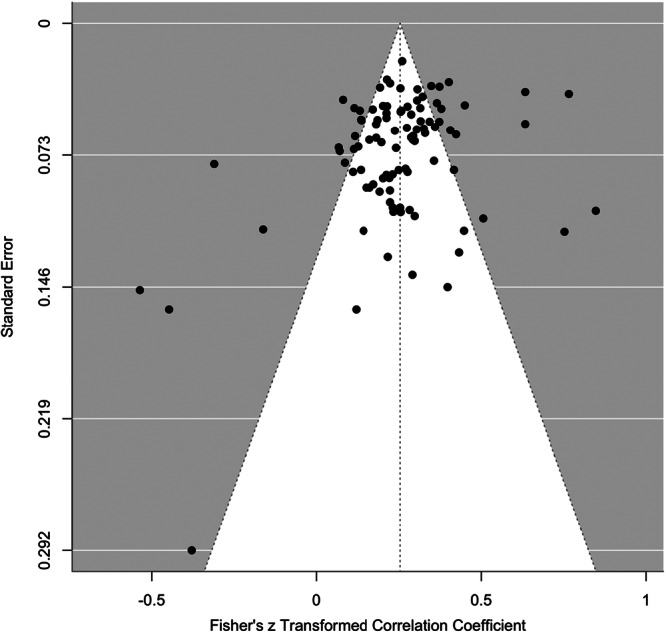
Funnel Plot Showing Publication Bias for the Association between Difficulties in Emotion Regulation and Aggression.

A multilevel model fit the data better than a single level model (χ^2^ (1) = 59.99, *p* < 0.001). An orchard plot in Figure 2 shows that there was a significant positive pooled association between aggression and difficulties in ER across 539 effect sizes from 100 studies (*r* = 0.248 [95% CI: 0.225–0.274], *p* < 0.001, *I* ^*2*^ = 89%), with most of the variance ( ~ 59%) attributable to effect sizes within studies. The common language effect size suggests that for any two people selected at random, a person with a higher value of aggression will have a higher value on difficulties in ER 58% of the time. Egger's test was significant (*z* = 3.13, *p* = 0.001), indicating evidence of publication bias. Trim and Fill imputed 94 effect sizes to improve the symmetry of the funnel plot (see Figure 5) and slightly increased the size of the effect in a single level model (*r* = 0.308 [95% CI: 0.296–0.325]). The PET slope was significant (*p* = 0.010), indicating evidence of small study effects. The bias corrected PEESE model showed a significant positive association (*r* = 0.281 [95% CI: 0.247–0.316]), indicating that even after adjusting for potential publication bias, a small‐to‐moderate positive effect remained. Cumulative meta‐analyses by year of publication demonstrated a stabilization of the effect from 2015 onwards (see Figure S1, Supporting Information). Greater distribution of *p*‐values closer to 0.01 compared to 0.05 suggests limited evidence of selective reporting of effects (see Figure S4, Supporting Information).

Type of aggression significantly moderated the relationship between difficulties in ER and aggression (χ^2^ (2) = 8.60, *p* = 0.013). The association for physical aggression (*n* = 177 effects from 58 studies: *r* = 0.233 [95% CI: 0.203–0.263]; *I* ^*2*^ = 81%) was not significantly different (*p* = 0.250) to sexual aggression (*n* = 26 effects from 10 studies: *r* = 0.204 [95% CI: 0.122–0.275]; *I* ^*2*^ = 81%), but did differ (*p* = 0.010) compared to other types of aggression (*n* = 336 effects from 78 studies: *r* = 0.268 [95% CI: 0.237–0.299]; *I* ^*2*^ = 91%). There was no significant difference in the strength of association for sexual aggression versus other types of aggression (*p* = 0.068). The target of aggression also significantly moderated the relationship between difficulties in ER and aggression (χ^2^ (1) = 15.54, *p* < 0.001), with a weaker association for intimate partners (*n* = 174 effects from 47 studies: *r* = 0.199 [95% CI: 0.170–0.227]; *I* ^*2*^ = 67%), versus other targets (*n* = 365 effects from 56 studies: *r* = 0.284 [95% CI: 0.253–0.315]; *I* ^*2*^ = 91%). Motivation for aggression significantly moderated the relationship (χ^2^(1) = 18.56, *p* < 0.001. The strength of the association was weaker for proactive (*n* = 9 effects from 8 studies: *r* = 0.222 [95% CI: 0.164–0.279]; *I* ^*2*^ = 52%) than reactive aggression (*n* = 10 effects from 9 studies: *r* = 0.404 [95% CI: 0.313–0.487]; *I* ^*2*^ = 85%). Gender did not significantly moderate the relationship (χ^2^(1) = 1.70, *p* = 0.191). The strength of the associations was similar for males (*n* = 239 effects from 38 studies: *r* = 0.231 [95% CI: 0.189–0.273]; *I* ^*2*^ = 88%) and females (*n* = 98 effects from 22 studies: *r* = 0.192 [95% CI: 0.154–0.229]; *I* ^*2*^ = 65%).

#### 5.4.4 Difficulties in Emotion Regulation (DERS) Subscales

**Figure 6:**
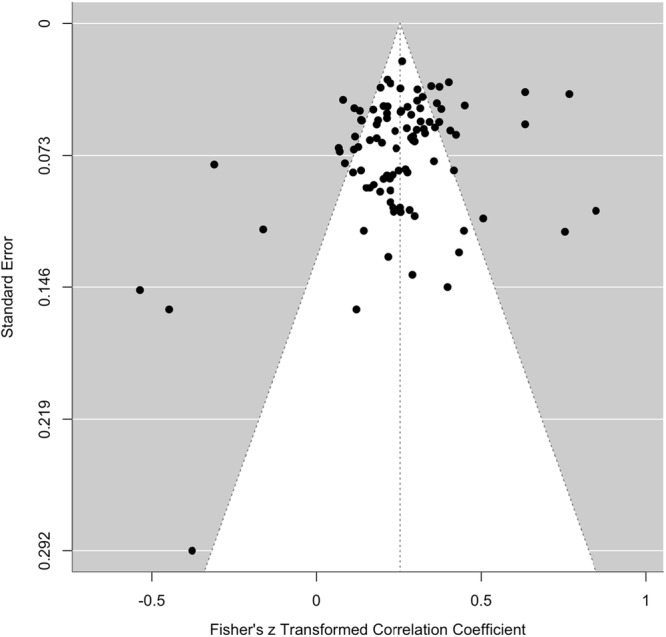
Funnel plot showing publication bias for the association of subscales of the difficulties in emotion regulation scale (DERS) and aggression.

When including only effects sizes based on the DERS, a multilevel model was a better fit of the data than a single level model (χ^2^(1) = 47.01, *p* < 0.001). There was a significant positive association of difficulties in emotion regulation subscales with aggression (*n* = 425 effect sizes from 40 studies: *r* = 0.230 [95% CI: 0.200–0.260], *p* < 0.001; *I* ^*2*^ = 87%) with the majority of variance ( ~ 64%) attributable to effect sizes within studies. The common language effect size suggests that for any two people selected at random, a person with a higher value of aggression will have a higher value on difficulties in emotional regulation subscales 57% of the time. Egger′s test was not significant (*z* = 1.84, *p* = 0.065), for a funnel plot see Figure 6.

**Figure 7:**
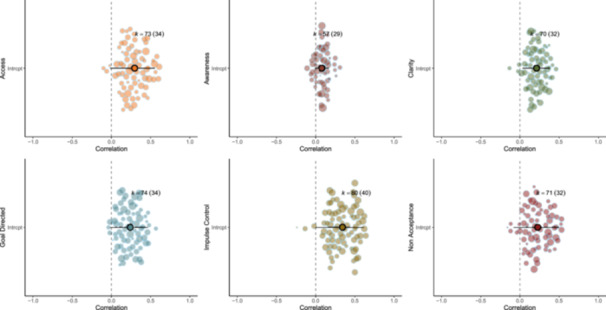
Orchard plot showing the meta‐analytic mean and individual effect sizes scaled by their precision for the moderator analysis on DERS subscales. [Color figure can be viewed at wileyonlinelibrary.com]

There was a significant moderation effect of DERS subscale (χ^2^ (5) = 145.80, *p* < 0.001). All subscales were significantly and positively associated with aggression (for an orchard plot see Figure 7): non‐acceptance (*n* = 71 effects from 32 studies: *r* = 0.229 [95% CI: 0.189–0.267]; *I* ^*2*^ = 86%); goal directed (*n* = 74 effects from 34 studies: *r* = 0.240 [95% CI: 0.207–0.272]; *I* ^*2*^ = 81%); awareness (*n* = 57 effects from 29 studies: *r* = 0.080 [95% CI: 0.057–0.104]; *I* ^*2*^ = 30%); access to ER strategies (*n* = 73 effects from 34 studies: *r* = 0.295 [95% CI: 0.257–0.331]; *I* ^*2*^ = 86%); clarity (*n* = 70 effects from 32 studies: *r* = 0.210 [95% CI: 0.184–0.236]; *I* ^*2*^ = 67%); impulse control difficulties (*n* = 80 effects from 40 studies: *r* = 0.343 [95% CI: 0.298–0.386]; *I* ^*2*^ = 89%). Specifically, associations were stronger for impulse control difficulties compared with goal directed (*p* < 0.001), access to ER strategies (*p* = 0.008), emotional awareness (*p* < 0.001), clarity (*p* < 0.001), and non‐acceptance (*p* < 0.001). Associations were stronger for access to ER strategies compared with goal directed (*p* = 0.014), emotional awareness (*p* < 0.001), and clarity (*p* < 0.001). Associations were stronger for non‐acceptance compared with emotional awareness (*p* < 0.001), and clarity (*p* < 0.001). Associations were stronger for goal‐directed compared with emotional awareness (*p* < 0.001). All other contrasts were non‐significant.

### 5.5 Guidance for Statistical Power

Based on PET‐PEESE bias corrected estimates, to reliably detect an association (one‐tailed, alpha = 0.05) for adaptive ER strategies (*r* = −0.106), approximately 696 participants are needed for 80% power, and 931 participants for 90% power; for maladaptive strategies (*r* = 0.355), 59 participants are needed for 80% power, and 79 participants for 90% power; and for difficulties in ER (*r* = 0.281), 97 participants are needed for 80% power, and 129 participants are needed for 90% power.

### 5.6 Quality Assessment

A summary of quality assessment of all papers included in this review can be seen in Table S4 in Supporting Information One. All the studies clearly reported their aims and objectives, had an appropriate study design, clearly defined the population of choice, used appropriate methods to recruit their chosen sample, used validated measures of ER and aggression, selected appropriate variables appropriate to the study aims, accurately reported descriptive statistics, reported results from all analyses stated in the methods, and included discussion and conclusion sections that were justified by the results. Most studies had a replicable methods section except two (Watkins et al. 2016 Caprara et al. 2014). Ten studies stated that measures were taken to address and categorize non‐responders, and 40 studies showed little risk of non‐response bias. Only twenty studies reported a power analysis, with several studies including small sample sizes. Eighty‐five studies clearly stated they had no funding sources or conflicts of interest, but nine studies did not state whether they had gained ethical approval or informed consent.

## 6 Discussion

We conducted a systematic review and meta‐analysis of the relationship between ER and aggression, with 171 studies from 137 articles published in peer‐reviewed journals between 2007 and 2024 identified for inclusion. In total, *N* = 252,605 participants and 918 effect sizes were included in a series of multilevel, random‐effects meta‐analyses. Publication years – with all articles published in the past 18 years, and 85% of all articles published in the past 10 years – speak to the burgeoning attention that ER research has attracted in relation to aggression.

Results of multilevel models showed that greater use of so‐called adaptive ER strategies was associated with lower aggression, while greater use of so‐called maladaptive strategies was associated with heightened aggression. Interestingly, the association of adaptive ER strategies with aggression was smaller in size than that of maladaptive strategies, and a non‐significant association was revealed after adjusting for small study effects using PET. These contrasting outcomes likely reflect both conceptual and methodological factors. The use of suppression, a maladaptive strategy, involves efforts to suppress experiential, behavioural, and physiological responses associated with an ongoing emotion, whereas adaptive strategies like cognitive reappraisal might produce more subtle effects on aggression and operate more indirectly through cognitive reframing and modifying appraisals (Gross 2002). Further, although most studies suggest that cognitive reappraisal is helpful for reducing general distress, adaptive strategies are less strongly related to externalizing disorders compared to maladaptive strategies like rumination and suppression (Aldao et al. 2010), and expressive suppression can be more helpful in some contexts, especially during brief periods and social interactions (Webb et al. 2012) Sheppes and Gross (2011). also highlight that while reappraisal is an antecedent focused strategy, it might also be used to modify an appraisal after emotional response tendencies develop, where its impact on more intensely felt emotions might be limited.

Associations for both adaptive and maladaptive strategies were moderated by type of aggression, with the use of adaptive strategies associated with lower levels of physical and ‘other’ types of aggression, while the use of maladaptive strategies was associated with increased use of all types of aggression, with the strongest effect size for ‘other’, compared to physical or sexual. For adaptive strategies, the association with sexual aggression was non‐significant, but this was based on effect sizes extracted from a single study in an all‐male community sample and should be interpreted with a degree of caution. Difficulty regulating emotions (e.g., reduced access to ER strategies or greater impulse control difficulties) was also associated with greater levels of aggression, and a moderator analysis showed that the association was smaller for physical compared to ‘other’ types of aggression.

Our results are consistent with theoretical perspectives, including the GAM (Anderson and Bushman 2002), and the *I* ^3^ model (Finkel and Hall 2018), which both highlight the importance of effective emotion regulation strategies for managing aggression. Except for adaptive ER strategies, where the association with sexual aggression was based on a single study, physical and sexual aggression were both associated with greater use of maladaptive strategies, and greater difficulties in ER. Our finding for sexual aggression is consistent with theoretical (e.g., the Integrated Theory of Sexual Offending Ward et al. 2006), and empirical (Gillespie et al. 2012; Gillespie et al. 2018a, 2018b), work suggesting that people who have sexually offended show problematic ER.

Our findings are also consistent with the Process Model of Emotion Regulation (Gross and John 2003; Sheppes et al. 2015), and Gratz and Roemer (2004) model of difficulties in ER. For example, in the context of the Process Model, our findings showed that increased use of strategies typically labelled as maladaptive and associated with adverse outcomes including suicidality (Rogier et al. 2024), eating disorders (Smith et al. 2018), and mood disorders and alcohol misuse (Aldao et al. 2010 Nolen‐Hoeksema and Watkins 2011), was associated with greater aggression. On the other hand, however, use of strategies typically labelled adaptive was associated with lower aggression. Earlier work suggests that increased use of response‐focused strategies, including suppression and avoidance, might help to reduce aggression by preventing retaliation to anger (Sheppes et al. 2015), but our findings instead support the notion that suppression and avoidance are habitually unhelpful strategies for managing aggression (Navas‐ Casado et al. 2023 Roberton et al. 2014). However, the extent to which different strategies might be more readily accessible in some circumstances and allow for a more flexible pattern of ER (Aldao et al. 2015) is worthy of further investigation.

In the context of Gratz and Roemer (2004) model, each of the DERS subscales was significantly and positively associated with aggression, with the strongest effect sizes observed for impulse control difficulties, consistent with earlier work highlighting the importance of self‐control problems in predicting violent, drug, and sexual offences (Epper et al. 2022). Indeed, brain imaging studies have shown greater activity in brain regions associated with impulse control and top‐down ER processes, including lateral and medial prefrontal cortex, when participants were subjected to provocation, highlighting the importance of inhibitory processes (Denson et al. 2009 Krämer et al. 2008). These findings highlight the importance of using effortful, cognitively demanding ER strategies to reappraise and reinterpret situations that can give rise to hostile thoughts and angry impulses, and ultimately lead to reactive aggression (Davidson et al. 2000; Denson 2015).

Other moderating factors identified in our analyses included the target of aggression, the motivation for aggression, and the gender of the aggressor. When considering the target of aggression, although the use of maladaptive strategies and difficulties in ER were both associated with aggression towards intimate partners and others, effect sizes tended to be stronger for aggression directed toward other targets. Significant associations with IPV are nonetheless supported by earlier meta‐analytical findings (Maloney et al. 2023) and suggest that interventions focused on resolving difficulties associated with impulse control, emotional acceptance, clarity, and awareness might help to reduce IPV (Chambers et al. 2009).

The finding that ER difficulties were more strongly associated with reactive than proactive motivations was largely unsurprising. Reviews of experimental and fMRI studies support this position (Davidson et al. 2000; Fanning et al. 2017; Katja et al. 2020; Gillespie et al. 2018), with reactive aggression typically associated with differences in activity and connectivity between brain regions associated with the processing of reward and threat, ER, and cognitive control. Interestingly, motivation for aggression did not significantly moderate the relationship of maladaptive strategies with aggression, suggesting that the distinction between reactive and proactive aggression with respect to ER processes is more nuanced than commonly believed (Garofalo 2022; Howard et al. 2009; Smeijers et al. 2020). While it makes conceptual sense that difficulties in controlling emotions and behaviour when distressed are more strongly linked to reactive than proactive aggression, it is interesting to note that use of maladaptive ER strategies was linked to both reactive and proactive aggression with similar strength. It is likely that certain maladaptive ER strategies like rumination could be important for forms of aggression that appear to be planned and calculated but are often inaccurately considered unrelated to ER problems. No studies in our review examined the relationship between so‐called adaptive strategies and proactive or reactive aggression highlighting a gap in the literature.

### 6.1 Strengths and Limitations

Our review had several strengths, including a comprehensive search strategy and duplication of selection of papers and data extraction by two authors, and the use of tests including PET‐PEESE, cumulative meta‐analysis, and analysis of *p*‐curves, but some limitations must be considered. First, it was not always possible to examine the associations of ER separately for different types of aggression, and effect sizes categorized as ‘other’ often included items relating to physical and psychological aggression. Second, our review only included studies that used self‐report measures, with 98% of these using a cross‐sectional design. Although other measurement techniques have been used to examine the ability to up‐ or down‐regulate emotions, or the ability to reappraise or suppress an emotional response, these studies often rely on brain imaging techniques that should be synthesized using alternative methods. The over‐reliance on cross‐sectional studies poses difficulties for understanding directional associations and suggests a need for future longitudinal work. Other methodologies, including daily dairies studies (Bolger et al. 2003; Barta et al. 2012), or ecological momentary assessment (Murray et al. 2022), represent useful data collection methods that might offer a finer understanding of these relationships beyond trait‐based measures. Third, restricting inclusion to reports written in English and published in peer‐reviewed journals may reduce the generalizability of the findings, and might be vulnerable to publication bias. Although Egger′s test was significant for adaptive and maladaptive strategies, studies imputed to reduce asymmetry in the funnel plots had a negligible impact on effect sizes. Other tests have also been recommended and are included in our review to account for publication bias, but these tests are also subject to some criticism, including PET‐PEESE (Stanley 2017), and the *p*‐curve procedure (Morey and Davis‐Stober 2025). It should also be noted that these tests cannot fully account for missing studies that live unseen in the file drawer (Carter et al. 2019). Finally, the use of some strategies was assessed more often than others, and it was notable that only one study measured the relationship between use of adaptive strategies and sexual aggression (Marín‐Morales et al. 2022). Future research should therefore examine ER strategy use in relation to sexual aggression to support the design of evidence‐based interventions that might help to reduce risk of sexual offending (Gillespie et al. 2018a, 2018b). An assessment of methodological quality also highlighted several limitations of studies included in the review. For example, very few studies were either pre‐registered or included a power calculation, and only a quarter reported undertaking measures to address non‐response bias.

### 6.2 Clinical Implications

Our results are uncertain with regard to recommending the use of interventions that aim to equip people with more adaptive strategies for regulating emotions to reduce aggressive behaviour. For example, reappraisal represents an essential component of many forms of psychotherapy (Berking et al. 2008; Beck 2011) and can be operationalized via a wide range of cognitive change tactics to down‐regulate negative emotion (Denny et al. 2023), but our meta‐analysis revealed only a small effect size, and the effect was non‐significant following PET. In addition to specific ER strategies, more holistic approaches, such as mentalization‐based therapy (Fonagy et al. 2025), which target individuals′ ER skills more broadly, including emotional awareness, acceptance and self‐reflection, may prove beneficial in reducing aggression, and specifically reactive aggression. Similarly, it has been proposed that mindfulness‐based interventions, which have been shown to be effective for reducing anger and aggression (O'Dean et al. 2025), might help to improve ER and reduce reoffence risk in people with a history of sexual and violent offences (Gillespie et al. 2012), and could help to reduce aggression in community samples (Garofalo and Wright 2017).

## 7 Conclusion

Our results showed that the use of so‐called maladaptive strategies, and difficulties in ER, were both associated with increased aggression, but the use of so‐called adaptive strategies was associated with lower aggression with a small effect size that was non‐significant following PET. These effects were variably moderated by the type and target of aggression, motivation for aggression, and gender. Effect sizes tended to be stronger for physical or ‘other’ types of aggression compared to sexual aggression, and were weaker when intimate partners were targets of aggression. Difficulties in ER were, unsurprisingly, more strongly associated with reactive aggression. When considering the different subscales of the DERS, impulse control difficulties had the strongest relationship with aggression. More research is needed to better understand associations with sexual aggression, while longitudinal studies and the use of EMA methodology would help to understand directional associations and the role of momentary factors. The findings reported here provide indirect support for interventions that aim to prevent physical, sexual, and other types of aggression with a focus on exploring and challenging angry dispositions and developing skills to manage impulsivity.

## Conflicts of Interest

The authors declare that they have no known competing financial interests or personal relationships that could have appeared to influence the work reported in this paper.

## Supporting information

Supplemental Material.

## Data Availability

The data that support the findings of this study are openly available in Open Science Framework at https://osf.io/t7wa6/.
