# Supplementary material for: Emotion Regulation and Aggression: A Systematic Review and Meta‐Analysis
Source: Aggress Behav. 2025 Dec 22;52(1):e70055. doi: 10.1002/ab.70055 (PMC12720225; doi:10.1002/ab.70055)
Supplement: Supplementary file 1 — Supplemental Material. [file AB-52-e70055-s001.docx]

**Supplemental Material**

Emotion regulation and aggression: A systematic review and meta-analysis

Kimberley Smith^1^, Andrew Jones^2^, Natasha Daly^1^, Helena Widdrington^1,^

Carlo Garofalo^3^, Steven M. Gillespie^1^

^1^Department of Primary Care and Mental Health, University of Liverpool, Liverpool, UK

^2^School of Psychology, Liverpool John Moores University, Liverpool, UK

^3^Department of Philosophy, Social Sciences, and Education, University of Perugia, Perugia, Italy

*Authors note:* Correspondence concerning this article should be addressed to Dr Steven M. Gillespie, Department of Primary Care and Mental Health, University of Liverpool, Liverpool, UK, L69 7ZA, e-mail: [steven.gillespie@liverpool.ac.uk](mailto:steven.gillespie@liverpool.ac.uk)

**Table S1**
Characteristics of the studies included in the meta-analyses.

| Author, year | Participants, sample type, location | Age (mean, SD) [range],  Gender | Ethnicity or nationality | Measure of Emotion Regulation (scale) | Measure of Aggression (scale) |
| --- | --- | --- | --- | --- | --- |
| El-Lim et al., 2022 | 313,  Students, US | (19.61, 3.51), Mixed |  | Reappraisal (ERQ)  Rumination (RRQ) | Physical, Verbal, Hostility,  Anger (BPAQ) |
|  | 460,  Community, US | (32.78, 11.79), Mixed | White (70.7%), Asian (8.95), African American (8.3%), Hispanic (7%),  Other (5.1%) | Suppression & Reappraisal (ERQ)  Rumination (RRQ)  Anger rumination (ARS) | Physical, Verbal, Hostility,  Anger (BPAQ) |
| Mellat et al., 2023 | 700,  Students, Iran | [20-60], Mixed |  | Difficulties in ER* (DERS) | Aggression total score (BPAQ-SF) |
| Anestis et al., 2009 | 200,  Students, US | (18.23, 1.09) [16-25], Mixed | White (68%), African American (15%), Hispanic (9.5%), Asian (2.5%), Other (3%) | Anger Rumination (ARS)  Rumination (CERQ) | Physical, Verbal, Hostility,  Anger (BPAQ) |
| Barlett, 2022 | 216,  Community, US | (36.07, 10.29), Mixed | White (87%) | Anger Rumination (TAR)  Rumination (RRQ) | Physical (BPAQ)  Proactive & Reactive (APBQ) |
| Bell et al., 2022 | 199,  Students, Canada | (19.03, 1.31), Female | White (69.2%), African American (18.8%), Hispanic (3.4%), Asian (3.4%), Other (5.1%) | Difficulties in ER* (DERS) | Physical & psychological IPV (CTS2) |
| Bilton et al., 2016 | 162,  Students, US | (18.92, 2.05) Male | White (81.4%), African American (8.9%), Hispanic (2.2%), Asian (3.2%), Other (4%) | Non-acceptance,  Goal-direct behaviour, Control difficulties,  Emotional awareness, Emotional clarity, Emotion regulation strategies (DERS) | Physical IPV (CTS2) |
|  | 117,  Students, US | (18.92, 2.05) Male | White (81.4%), African American (8.9%), Hispanic (2.2%), Asian (3.2%), Other (4%) | Non-acceptance,  Goal-direct behaviour, Control difficulties,  Emotional awareness, Emotional clarity, Emotion regulation strategies (DERS) | Psychological IPV (CTS2) |
|  | 326,  Students, US | (18.92, 2.05) Female | White (81.4%), African American (8.9%), Hispanic (2.2%), Asian (3.2%), Other (4%) | Non-acceptance,  Goal-direct behaviour, Control difficulties,  Emotional awareness, Emotional clarity, Emotion regulation strategies (DERS) | Physical IPV (CTS2) |
|  | 346, Students, US | (18.92, 2.05) Female | White (81.4%), African American (8.9%), Hispanic (2.2%), Asian (3.2%), Other (4%) | Non-acceptance,  Goal-direct behaviour, Control difficulties,  Emotional awareness, Emotional clarity, Emotion regulation strategies (DERS) | Psychological IPV (CTS2) |
| Borders et al., 2007 | 285,  Students, US | (19.8, -) [17-50], Mixed | White (55%), Asian (25%), Hispanic (12%)  African American (3%)  Other (5%) | Rumination (RRQ) | Aggression total score (BPAQ) |
| Borders et al., 2010 | 464,  Students, US | (19.7, -), Mixed | White (50%), Asian (19%), Hispanic (8%)  African American (7%)  Other (13%) | Rumination (RRQ) | Physical, Verbal, Hostility,  Anger (BPAQ) |
|  | 211,  Community, US | (31.8, 11.8),  Mixed | White (80%), Asian (5%)  Hispanic (3%), African American (5%), Other (5%) | Rumination (RRQ) | Physical, Verbal, Hostility,  Anger (BPAQ) |
| Borders et al., 2011 | 304,  Students, US | (20, -) [18-44], Mixed | Asian (25%), Hispanic (35%), African American (25%), Other (15%) | Anger Rumination (ARS) | Physical, Verbal, Hostility, Anger (BPAQ) |
| Buyuks et al., 2023 | 113,  Clinical (Schizophrenia & Bipolar), Turkey | Schizophrenia (40.4, 11.3) & Bipolar (35.9, 10.1),  Mixed |  | Non-acceptance,  Goal-direct behaviour, Control difficulties,  Emotional awareness, Emotional clarity, Emotion regulation strategies (DERS) | Physical, Verbal, Hostility, Anger (BPAQ) |
| Dilek et al., 2021 | 714,  Community, Turkey | (25.16, -) [18-64],  Mixed | Turkish | Rumination (RTSQ) | Aggression total score (AES) |
| Chatzimike‑Levidi & Collard, 2023 | 351,  Community, Australia | (26.56, 10.53) [18-69], Mixed | American (29.3%), Australian (22.5%)  European (13.7%) | Angry Afterthoughts  Thoughts of Revenge  Angry Memories  Understanding of causes (ARS) | Physical, Verbal, Hostility, Anger (BPAQ) |
| Hoover et al., 2021 | 124,  Clinical (Family therapy clinic), US | (40.71, 11.58) [22-72], Female | White (59.3%) Asian  (11.9%,), Hispanic (20%,), Black (9.1%), Other (7.2%,) | Difficulties in ER* (DERS) | Psychological IPV (CTS2) |
|  | 124,  Clinical (Family therapy clinic), US | (42.20, 11.82) [24-74], Male | White (59.3%), Asian (7.2%), Hispanic (14.5%), Black (13.6%), Other (11.8%) | Difficulties in ER* (DERS) | Psychological IPV (CTS2) |
| Demichelis et al., 2022 | 740,  Community, Australia | (34.77, 12.95) [18-67],  Mixed |  | Difficulties in ER* (DERS) | Physical, Verbal, Hostility, Anger (BPAQ) |
| Donahue et al., 2014 | 318,  Students, US | (21.35, 5.83) [18-67], Mixed | White (62.6%), Asian (5%), Hispanic (11%)  African American (16%)  Other (5.4%) | Non-acceptance,  Goal-direct behaviour, Control difficulties,  Emotional awareness, Emotional clarity, Emotion regulation strategies (DERS) | Physical (BPAQ) |
| Edwards et al., 2017 | 96,  Students, US | [18-38],  Mixed | White (15%), Asian (16%), Hispanic (40%)  African American (16%)  Other (8%) | Difficulties in ER* (DERS) | Impulsive aggression (IPAS) |
| Fresnics et al., 2017 | 201,  Students, US | (19.48, 1.47) [18-29], Mixed | White (73%), Asian (9%)  Hispanic (8%),  African American (5%)  Other (5%) | Anger rumination (ARS) | Aggression (EAS) |
| Garofalo et al., 2020 | 397,  Offenders, Italy | (40.26, 11.96), Male |  | Non-acceptance,  Goal-direct behaviour, Control difficulties,  Emotional awareness, Emotional clarity, Emotion regulation strategies (DERS) | Physical, Verbal, Hostility, Anger (BPAQ) |
|  | 324,  Community, Italy | (37.87, 12.06), Male | Italian | Non-acceptance,  Goal-direct behaviour, Control difficulties,  Emotional awareness, Emotional clarity, Emotion regulation strategies (DERS) | Physical, Verbal, Hostility, Anger (BPAQ) |
| Garofalo et al., 2016 | 153,  Offenders, Italy | (41.78, 11.84), Male | White (100%) | Non-acceptance,  Goal-direct behaviour, Control difficulties,  Emotional awareness, Emotional clarity, Emotion regulation strategies (DERS) | Physical, Verbal, Hostility, Anger (BPAQ) |
|  | 197,  Community, Italy | (38.88, 10.89), Male |  | Non-acceptance,  Goal-direct behaviour, Control difficulties,  Emotional awareness, Emotional clarity, Emotion regulation strategies (DERS) | Physical, Verbal, Hostility, Anger (BPAQ) |
| Garofalo et al., 2021 | 268,  Offenders, Italy | (37.36, 11.82), Male | Italian (46%), European (20%), African (23%),  Other (17%) | Difficulties in ER* (DERS) | Physical, Verbal, Hostility, Anger (BPAQ) |
|  | 521,  Community, Italy | (35.27, 15.99), Mixed | Dutch | Difficulties in ER* (DERS) | Reactive & proactive (RPQ) |
| Garofalo et al., 2018 | 221,  Offenders, Italy | (40.9, 9.40) [25-60], Male | Italian | Non-acceptance,  Goal-direct behaviour, Control difficulties,  Emotional awareness, Emotional clarity, Emotion regulation strategies (DERS) | Physical, Verbal, Hostility, Anger (BPAQ) |
|  | 245,  Community, Italy | (38.9, 10.1) [25-60], Male | Italian | Non-acceptance,  Goal-direct behaviour, Control difficulties,  Emotional awareness, Emotional clarity, Emotion regulation strategies (DERS) | Physical, Verbal, Hostility, Anger (BPAQ) |
| Garofalo et al., 2017 | 268,  Offenders, Italy | (37.36, 13.91), Male | Italian | Difficulties in ER* (DERS) | Aggression total score (BPAQ) |
| Gómez-Leal et., 2022 | 282,  Community, Spain | (22.02, 2.90) [18-30], Mixed |  | Reappraisal & Suppression (ERQ) | Aggression total score (BPAQ) |
| Grigorian et al., 2022 | 391,  Offenders, US | (33.37, 11.43), Male | White (60.4%), Hispanic (12.5%), Black (9.5%),  Other (3.1%) | Difficulties in ER* (DERS) | Psychological & Physical IPV (CTS2) |
| Grigorian et al., 2019 | 71,  Offenders, US | (30.34, 10.73), Female | White (73.2%), Hispanic (9.9%), Black (5.6%), Other (4.2%) | Non-acceptance,  Goal-direct behaviour, Control difficulties,  Emotional awareness, Emotional clarity, Emotion regulation strategies (DERS) | Psychological & Physical IPV (CTS2) |
| Guerra et al., 2017 | 610,  Students, US | (18.84, 0.78) [18-20],  Mixed | White (85.4%), Asian (9%), Other (5.6%) | Anger rumination (ARS) | Proactive & Reactive (APBQ) |
| Guzmán-González et al., 2016 | 369,  Students, Chile | (21.41, 2.26) [18=29], Female |  | Difficulties in ER* (DERS) | Physical (CTS2) |
|  | 239,  Students, Chile | (21.52, 2.15) [18-28],  Male |  | Difficulties in ER* (DERS) | Physical (CTS2) |
| Hasegawa et al., 2022 | 213,  Students, Japan | (19.76, 1.44) [18-25], Mixed | Japanese (99.5%), Vietnamese (.05%) | Rumination (RRS) | Aggressive behaviour (AS) |
| Hayes et al., 2021 | 110,  Students, US | (18.86, 1.17) [18-25], Female | White (67.6%), Hispanic (9.5%), Black (9.5%),  Other (6.8%) | Rumination (RRS)  Difficulties in ER* (DERS) | Dating & peer physical aggression (SRASMB) |
|  | 37,  Students US | (18.86, 1.17) [18-25], Male | White (67.6%), Hispanic (9.5%), Black (9.5%),  Other (6.8%) | Rumination (RRS)  Difficulties in ER* (DERS) | Dating & peer physical aggression (SRASMB) |
| He et al., 2022 | 397,  Students, China | (20.11, 1.40) [17-25], Mixed |  | Trait acceptance (AAQ-11) | Aggression total score (BPAQ) |
| Kirwan et al., 2019a | 102,  Students, US | [18-30+], Male | White (78.2%), Asian (3%), Middle Eastern (5.9%), African American (3%), Other (8%) | Difficulties in ER* (DERS)  Control difficulties (DERS) | Sexual assault preparation (SES) |
| Kirwan et al., 2019b | 334,  Community, US | (35.41, 11.08), Mixed | White (76.3%), Asian (8.4%), Black (10.5%)  American Indian (9%)  Other (6%) | Difficulties in ER* (DERS) | Physical & verbal (BPAQ)  IPV (RVS)  Sexual assault preparation (SES) |
| Lee et al., 2020 | 80,  Students, UK | (20.28, 2.65) [18-30], Male | White (92.5%) | Difficulties in ER* (DERS) | Physical & psychological IPV (SD)  Sexual assault preparation (SES-SF) |
|  | 80,  Students, UK | (20.28, 2.65) [18-30], Female | White (92.5%) | Difficulties in ER* (DERS) | Physical & psychological IPV (SD)  Sexual assault preparation (SES-SF) |
| Lewis et al., 2014 | 220,  Community, US | Average 54, Female | Mainly White | Rumination (RSQ) | Psychological IPV (CTS2) |
| Li et al., 2022 | 942,  Students, China | (20.3, 1.05) [17-26], Mixed |  | Anger rumination (ARS) | Physical (BPAQ) |
| Lilly et al., 2014 | 254,  Community, US | (31.78, 10.39), Female | White (45.84), African American (41.1%) | Difficulties in ER* (DERS) | Psychological & Physical IPV (CTS2) |
| Logoz et al., 2023 | 428,  Community, US | (43.9, 15.3), Male | German (69.95%) | Reappraisal & suppression (ERQ) | Aggression total score (BPAQ) |
| Long et al., 2014 | 81,  Clinical (substance use facility), US | (42, 1.24), [18-49], Mixed | White (7.4%), African American (85.2%)  Other (4.9%) | Difficulties in ER* (DERS) | Proactive & reactive (IPAS) |
| Mancke et al., 2017 | 95,  Clinical (Personality disorder), Germany | (28.45, 7.18) [18-49], Mixed |  | Difficulties in ER* (DERS) | Aggression total score (BPAQ) |
| Mansfield et al., 2009 | 49,  Clinical (anger management programme), US | 18+,  Male | White (64%), Hispanic (14%), African American (13%), Other (8%) | Emotional intelligence (TMMS) | Aggression total score (BPAQ) |
|  | 43,  Clinical (anger management programme), US | 18+,  Female | White (64%), Hispanic (14%), African American (13%), Other (8%) | Emotional intelligence (TMMS) | Aggression total score (BPAQ) |
| Martino et al., 2015 | 151,  Clinical (Personality disorder), Italy | (33.9, 9.8), Mixed |  | Difficulties in ER* (DERS)  Anger rumination (ARS) | Aggression total score (BPAQ) |
| Martino et al., 2018 | 91,  Clinical (Personality disorder), Italy | (32.8, 10), Mixed |  | Difficulties in ER* (DERS)  Anger rumination (ARS)  Rumination (RSQ) | Aggression total score (BPAQ) |
| Massa et al., 2017 | 271,  Community, US | (33.13, 10.73), Mixed |  | Rumination (RRQ) | Anger & Hostility (BPAQ)  IPV preparation (CTS2) |
| Miles et al., 2016 | 259,  Clinical (Veterans inpatient treatment), US | (30.80, 7.11), Male | White (68.5%), Asian (.8%), Hispanic (8.8%), African American (16.2%), Other (5.7%) | Non-acceptance,  Goal-direct behaviour, Control difficulties,  Emotional awareness, Emotional clarity, Emotion regulation strategies (DERS) | Impulsive and premediated (IPAS) |
|  | 220,  Clinical (Veterans inpatient treatment), US | (41.04, 10.39), Female | White (45.8%) Asian (.5%), Hispanic (3.2%), African American (45.5%,), Other (5%,) | Non-acceptance,  Goal-direct behaviour, Control difficulties,  Emotional awareness, Emotional clarity, Emotion regulation strategies (DERS) | Impulsive and premediated (IPAS) |
| Oliveros et al., 2021 | 145,  Students, US | (19.50, 1.59), [18-30], Male |  | Difficulties in ER* (DERS) | IPV (AIRS) |
|  | 475,  Students, US | (19.50, 1.59), [18-30], Female |  | Difficulties in ER* (DERS) | IPV (AIRS) |
| Ortiz et al., 2015 | 379,  Students, US | (18.52, 1.24), Female |  | Difficulties in ER* (DERS) | Psychological & physical IPV (CTS2) |
| Peters et al., 2015 | 823,  Students, US | (19.25, 2.51), [18-59], Mixed | White (85.9%) | Anger rumination (ARS) | Physical, Verbal,  Hostility, Anger (BPAQ) |
| Pickett et al., 2017 | 56,  Students, US | (20, -), Male | White (75%) | Difficulties in ER* (DERS) | Physical & Hostility (BPAQ) |
|  | 54,  Students, US | (20, -), Male | White (75%) | Difficulties in ER* (DERS) | Physical & Hostility (BPAQ) |
| Pollard et al., 2016 | 208,  Student couples, Switzerland | (21.68, 4.04), Male | Hispanic (85.1%), one Hispanic (12%), non-Hispanic (2.9%) | Difficulties in ER* (DERS)  Control difficulties (DERS) | Physical IPV (CTS2) |
|  | 208,  Student couples, Switzerland | (20.74, 3.51), Female | Hispanic (85.1%), one Hispanic (12%), non-Hispanic (2.9%) | Difficulties in ER* (DERS)  Control difficulties (DERS) | Physical IPV (CTS2) |
| Preston et al., 2020 | 368,  Students, US | (21.49, 5.48), Mixed | White (64.7%), Black (28.8%), Hispanic (1.6%),  Other (5%) | Difficulties in ER* (DERS) | Reactive & proactive (RPQ) |
| Price et al., 2014 | 245,  Community, US | (32.02, 10.35), Female | White (47.1%), African American (40.2%), Hispanic (3.7%),  Other (7.3%) | Difficulties in ER* (DERS) | Physical IPV (CTS2) |
| Pugliese et al., 2015 | 948,  Students, US | (19.70, 1.63) [18-46], Mixed | White (81.3%), Asian (9.5%), Hispanic (3.3%),  African American (3.9%),  Other (5.2%) | Anger rumination (ARS) | Physical, Verbal, Hostility (BPAQ) |
| Puhalla et al., 2020 | 76,  Students, US | (21.49, 4.14) [18-27], Mixed | White (51%), African American (23%), Asian (21%), Other (5%) | Difficulties in ER* (DERS) | Reactive & proactive (RPQ) |
| Quan et al., 2020 | 600,  Students, China | (20.51, 1.11), Mixed |  | Anger rumination (ARS) | Reactive (RPQ) |
| Quan et al., 2021 | 1115,  Students China | (20.31, 1.23) [17-26], Mixed |  | Anger rumination (ARS) | Aggression total score (BPAQ) |
|  | 942,  Students, China | (21.09, 1.07) [18-27], Mixed |  | Anger rumination (ARS) | Aggression total score (BPAQ) |
| Quan et al., 2019 | 437,  Students, China | (20.79, 1.07), Mixed | Chinese | Anger rumination (ARS) | Aggression total score (BPAQ) |
| Sanchez-Ruiz et al., 2018 | 252,  Students, Lebanese | (19.44, 1.77) [16-30], Mixed | Lebanese | Reappraisal (ERQ) | Aggression total score (BPAQ) |
| Scott et al., 2014 | 150,  Clinical (Personality disorder), US | (44.86, 10.42) [22-61], Mixed |  | Difficulties in ER* (DERS) | Physical & psychological IPV (CTS2) |
| Shamsipour et al., 2018 | 260,  Students, Iran | [18-57], Mixed |  | Maladaptive emotion regulation, self-blame, others blame, rumination, acceptance, catastrophizing (CERQ-short) | Aggression total score (BPAQ) |
| Shorey et al., 2015 | 67,  Students, US | (19.74, 2.42) [18-34], Male | White (86.6%) | Difficulties in ER* (DERS) | Physical & psychological IPV (CTS2) |
| Shorey et al., 2011a | 187,  Students, US | (19.4, 1.6, 1.47), Male | White (85%), African American (9.1%), Asian (2.1%), Other (3.8%) | Non-acceptance,  Goal-direct behaviour, Control difficulties,  Emotional awareness, Emotional clarity, Emotion regulation strategies (DERS) | Physical, psychological & sexual IPV (CTS2) |
|  | 253,  Students, US | (19.0, 1.3), Female | White (85%), African American (9.1%), Asian (2.1%), Other (3.8%) | Non-acceptance,  Goal-direct behaviour, Control difficulties,  Emotional awareness, Emotional clarity, Emotion regulation strategies (DERS) | Physical, psychological & sexual IPV (CTS2) |
| Shorey et al., 2011b | 145,  Students, US | (18.6, 1.67), Female | White (94.5%) | Difficulties in ER* (DERS) | Psychological IPV (CTS2) |
| Shorey et al., 2014 | 109,  Students, US | (18.44, .75), Male | White (79.9%), African American (9.4%)  Other (11.4%) | Experiential avoidance (AAQ-11) | Physical, psychological & sexual IPV (CTS2) |
| Sotelo et al., 2013 | 134,  Community, US | (32, 10.1), Male | White (26.7%), Asian (.8%), Hispanic (14.5%),  African American (54.2%),  Other (3.1%) | Rumination (DRS) | Physical IPV (CTS2) |
| Stappenback et al., 2016 | 158,  Students, US | (20.2, 1.8) [18-32], Male | White (42%), Asian (38%), African American (3%), Other (17%) | Non-acceptance,  Goal-direct behaviour, Control difficulties,  Emotional awareness, Emotional clarity, Emotion regulation strategies (DERS) | IPV (DRVQ) |
| Terzi et al., 2017 | 79,  Clinical (Personality disorder), Italy | (34, 10.4), Mixed |  | Difficulties in ER* (DERS) | Aggression total score (BPAQ) |
| Thiessen et al., 2018 | 782,  Students, Canada | (22.20, 7.51), Female |  | Difficulties in ER* (DERS) | Physical IPV (CTS2) |
|  | 484,  Students, Canada | (23.71, 7.96), Male |  | Difficulties in ER* (DERS) | Physical IPV (CTS2) |
| Trombetta et al., 2023 | 120,  Community, Switzerland | (33.8, 11.5) [20-77], Mixed |  | Difficulties in ER* (DERS) | Controlling behaviour IPV (CBS-R) |
| Turner et al., 2015 | 729,  Students, US | [18-24]. Mixed | White (86%), Asian (8.5%), Hispanic (2.7%),  Black (2.3%), Other (6.1%) | Anger rumination (ARS) | Reactive & proactive (RPQ) |
| Velotti et al., 2016 | 257,  Clinical (Psychiatric inpatient), Italy | (43.89, 13.74), Mixed |  | Non-acceptance,  Goal-direct behaviour, Control difficulties,  Emotional awareness, Emotional clarity, Emotion regulation strategies (DERS) | Aggression total score (BPAQ) |
|  | 617,  Community, Italy | (36.88, 13.11), Mixed |  | Non-acceptance,  Goal-direct behaviour, Control difficulties,  Emotional awareness, Emotional clarity, Emotion regulation strategies (DERS) | Aggression total score (BPAQ) |
| Wahlstrom et al., 2015 | 60,  Clinical (substance use facility), US | (34.4, 7.9),  Male | White (80%), Hispanic (8.3%), Black (6.7%),  Other (5%) | Non-acceptance,  Goal-direct behaviour, Control difficulties,  Emotional awareness, Emotional clarity, Emotion regulation strategies (DERS) | Physical IPV (CTS2) |
| Wang et al., 2018 | 464,  Students, China | (20.71, 1.59) [17-25], Mixed |  | Anger rumination (ARS) | Physical & verbal (BPAQ) |
| Wang et al., 2020 | 505,  Students, China | (20.12, 1.03) [17-24], Mixed |  | Anger rumination (ARS) | Reactive & proactive (RPQ) |
|  | 437,  Community, China | (20.05, 1.12) [17-26], Mixed |  | Anger rumination (ARS) | Reactive & proactive (RPQ) |
| Watkins et al., 2016 | 77,  Clinical (substance use facility), US | (32.95, 9.69) [18-55], Mixed | White (81.8%) | Non-acceptance,  Goal-direct behaviour, Control difficulties,  Emotional awareness, Emotional clarity, Emotion regulation strategies (DERS) | Physical & psychological IPV (CTS2) |
| Wei et al., 2020 | 578,  Community, China | [18+] 363 under 30 and 215 over 30, Male |  | Reappraisal & suppression (ERQ) | IPV preparation (IPV-GBM) |
| White et al., 2014 | 359,  Students, US | (19.13, 1.21) [18-23], Mixed | White (84.7%), Asian (10%), Other (5%) | Anger rumination (ARS) | Reactive & proactive (RPQ) |
| Yang, 2020 | 186,  Students, US | (19.4, 2.47) [18-37], Mixed | White (57%), African American (32%), Other (11%) | Difficulties in ER* (DERS) | Psychological & physical IPV (CTS2) |
| Garofalo et al., 2017 | 221,  Offenders, Italy/Netherlands | (40.9, 9.40), Male | Italian | Non-acceptance,  Goal-direct behaviour, Control difficulties,  Emotional awareness, Emotional clarity, Emotion regulation strategies (DERS) | Physical (BPAQ) |
| Cen et al., 2022 | 1203,  Students, China | (18.94, 0.96), Mixed |  | Hostile rumination (DRS) | Reactive & proactive (RPQ |
| Chen et al., 2020 | 462,  Students, China | (19.60, .807), Mixed |  | Reappraisal & suppression (ERQ) | Aggression total score (BPAQ) |
| Velotti et al., 2017 | 541,  Community, US | (19.42, 1.51) [18-32], Mixed | White (63%), Asian (4%),  Hispanic (9%), African American (17%), Other (8%) | Non-acceptance,  Goal-direct behaviour, Control difficulties,  Emotional awareness, Emotional clarity, Emotion regulation strategies (DERS) | Physical, Verbal, Hostility, Anger (BPAQ) |
|  | 284,  Community, Italy | (25, 3.49), [18-32], Mixed | Italian (97.5%) | Non-acceptance,  Goal-direct behaviour, Control difficulties,  Emotional awareness, Emotional clarity, Emotion regulation strategies (DERS) | Physical, Verbal, Hostility, Anger (BPAQ) |
| Espirito-Santo et al., 2022 | 326,  Community, Portugal | (75.12, 8.78) [60-96], Mixed |  | Difficulties in ER* (DERS) | Physical, Verbal, Hostility, Anger (BPAQ) |
| Avnaim et al., 2022 | 102,  Clinical IPV programme, US | Female (30.53, 8.64), Male (32.49, 10.78), Mixed | F, M White (31.4%, 32%)  F, M Asian (5.9%, 4%)  F, M Hispanic (3.9%, 10%), F, M African American (39.2%, 44%), F, M Other (19.6%, 6%) | Difficulties in ER* (DERS) | Physical IPV (CTS2)  Emotional abuse (MMEA-SF) |
| Peled et al., 2010 | 226,  Students, Canada | (19.7, 3.08) [17-45], Mixed | Canadian (62%) | Anger rumination (SAIR) | Overt aggression (FFAM) |
| Orozco-Vargas et al., 2021 | 385  Community, Mexico | (32.57, 8.50), [18-57], Male | \|  \|  \| \| --- \| --- \| \|  \|  \| | Goal-direct behaviour, Control difficulties & Emotion regulation strategies (DERS)  Rumination, acceptance, re-focus on planning, and positive reappraisal (CERQ) | IPV (VSSI) |
|  | 435,  Community, Mexico | (32.57, 8.51) [18-57]  Female |  | Goal-direct behaviour, Control difficulties & Emotion regulation strategies (DERS)  Rumination, acceptance, re-focus on planning, and positive reappraisal (CERQ) | IPV (VSSI) |
| Tull et al., 2007 | 113,  Community, US | (26.41, 5) [18-65], Male | White (59%), Asian (2%)  Hispanic (11%), African American (12%)  Other (11%) | Experiential avoidance & Emotional in expressivity (AAQ) | Aggressive behaviour (BDHI) |
| Fern et al., 2023 | 460,  Community, Australia | (31.96, 11.02) [18-68], Mixed |  | Thoughts of revenge (ARS) | Overt aggression (LHAS-S-A) |
| Han et al., 2020 | 371,  Students, China | [18-20], Mixed |  | Rumination (RRS) | Aggression total score (BWAQ) |
| Hasegawa et al., 2022 | 201,  Students, Japan | (20.20, 2.83) [18-51], Mixed |  | Brooding & Reflection (RRS) | Aggression (AE) |
| Hosie et al., 2021 | 129,  Offenders, Australia | (33.51, 8.69) [18-62], Male | Australian (69.8%) | Non-acceptance,  Goal-direct behaviour, Control difficulties,  Emotional awareness, Emotional clarity, Emotion regulation strategies (DERS) | Overt aggression (LHAS-S-A) |
| Hosie et al., 2022 | 129,  Offenders, Australia | (33.54, 8.67) [18-62], Male | Australian (69%) | Angry Afterthoughts  Thoughts of Revenge  Angry Memories  Understanding of causes (ARS) | Overt aggression (LHAS-S-A) |
| Maxwell et al., 2007 | 376,  Athletes,  China | (21.47, 5.52) [18-45], Mixed | British | Angry Afterthoughts  Thoughts of Revenge  Angry Memories  Understanding of causes (ARS) | Aggression (SAS) |
|  | 308,  Athletes, China | (20.80, 2.54) [18-45], Mixed | Chinese | Angry Afterthoughts  Thoughts of Revenge  Angry Memories  Understanding of causes (ARS) | Aggression (SAS) |
| Rogier et al., 2019 | 380,  Community, Italy | Male (31, 11.41), Female (28.50, 9.88) [18-58], Mixed |  | Reappraisal & suppression (ERQ) | Physical, Verbal, Hostility, Anger (BPAQ) |
| Lin et al., 2020 | 69,  Clinical (Gaming disorder), Taiwan | (25.23, 4.20), Mixed |  | Emotional concealment, emotional adjustment & emotional tolerance (ASQ) | Hostility (BDHIS-SF) |
| Ray et al., 2020 | 409,  Community, US | (31.66) [18-73], Male | White (76.8%), Asian (8.3%), Hispanic (4.6%)  African American (1.7%)  Other (2%) | Control difficulties (DERS) | Aggression (SRBSR) |
| Li et al., 2019 | 346,  Students, China | (19.87, .89), Mixed |  | Anger rumination (ARS) | Aggressive behaviour (ASR) |
|  | 811,  Students, China | Freshman-Seniors, Mixed |  | Anger rumination (ARS) | Physical & Verbal (BPAQ) |
| Caprara et al., 2014 | 150  Community, Italy | (19, 0.83) [18-20], Male |  | Hostile Rumination (DRS) | Aggression and Violence (VAS) |
|  | 164,  Community, Italy | (19, .083) [18-20], Female |  | Hostile Rumination (DRS) | Aggression and Violence (VAS) |
| Sarrate-Costa et al., 2023 | 47,  Offenders, Spain | (42.30, 1.73), Male |  | Anger rumination (ARS) | Reactive & proactive (RPQ) |
|  | 36,  Community, Spain | (37.34, 1.74), Male |  | Anger rumination (ARS) | Reactive & proactive (RPQ) |
| Ireland et al., 2020 | 442,  Community, UK | (25.6, 8) [18-66], Mixed |  | Difficulties in ER* (DERS) | Reactive & proactive (RPQ) |
| Holley et al., 2017 | 168,  Students, US | (22.62, 6.2) [18-57], Mixed | White (36.5%), Asian (15%), Hispanic (25.1%)  African American (9%)  Other (14.4%) | Difficulties in ER* (DERS) | Aggression behaviour (MCVI) |
| Conzemius et al., 2021 | 132,  Students, US | (19.19, 1.26), Male | White (82.6%), Black (10.2%), Asian (2.4%), Hispanic (1.8%)  Other (3%) | Difficulties in ER* (DERS) | Physical & psychological IPV (CTS2) |
| Velotti et al., 2020 | 203,  Community, Italy | (38.68, 11.98), Male |  | Difficulties in ER* (DERS) | Physical, Verbal, Hostility, Anger (BPAQ) |
|  | 182,  Offenders, Italy | (37.12, 13.42), Male |  | Difficulties in ER* (DERS) | Physical, Verbal, Hostility, Anger (BPAQ) |
| Kayha et al., 2019 | 253,  Community, Turkey | (26.48, 6.58), Female |  | Non-acceptance,  Goal-direct behaviour, Control difficulties,  Emotional awareness, Emotional clarity, Emotion regulation strategies (DERS) | Physical, psychological & sexual IPV (CTS2) |
| Marín-Morales et al., 2022 | 29,  Community, Spain | (38.28, 8.24), Male |  | Cognitive reappraisal, suppression, self-blame, acceptance, rumination, positive refocusing, refocus on planning, positive reappraisal, putting into perspective, others-blame & catastrophizing (CERQ) | Physical, psychological & sexual IPV (CTS2) |
|  | 26,  Offenders, Spain | (41.19, 9.71),  Male |  | Cognitive reappraisal, suppression, self-blame, acceptance, rumination, positive refocusing, refocus on planning, positive reappraisal, putting into perspective, others-blame & catastrophizing (CERQ) | Physical, psychological & sexual IPV (CTS2) |
| Andrade et al., 2017 | 700,  Community, Mexico | (38.6, 12.42),  Mixed |  | Angry Afterthoughts  Thoughts of Revenge  Angry Memories  Understanding of causes (ARS) | Physical, Verbal, Hostility, Anger (BPAQ) |
| Hsieh & Chen, 2017 | 80,  Students, Spain | (21.57, 1.75), Mixed |  | Emotion Regulation (NMRS) | Aggression total score (BPAQ) |
| Toro et al., 2020 | 630,  Community, Columbia | (31.07, 11.27) [18-63], Mixed |  | Anger rumination (ARS) | Reactive & proactive (RPQ) |
| Toro et al., 2019 | 520, Community, Columbia | (26.32, 6.25), Mixed |  | Anger rumination (ARS) | Reactive & proactive (RPQ) |
| Kuzyk et al., 2022 | 783, Community, US | (41, 12.26) [18-65], Mixed | Black (97%) | Non-acceptance,  Goal-direct behaviour, Control difficulties,  Emotional awareness, Emotional clarity, Emotion regulation strategies (DERS) | Aggression (BQ-S) |
| Caprara et al., 2013 | 340, Community, Italy | (21, .82) [20-22], Mixed |  | Hostile Rumination (DRS) | Engagement in violent and aggression (EVA) |
| Trombetta & Rollè, 2014 | 139,  Community, Italy | (34.2, 11.87) [20-76], Mixed |  | Difficulties in ER* (DERS) | Physical IPV (CTS2) |
| Moroń et al., 2023 | 482, Community, Poland | (23, 3.10), Mixed |  | Suppression and Cognitive Appraisal (ERQ) | Proactive & reactive overt aggression (PCS) |
| Fahmy & Dardis., 2024 | 598, Students, US | (19.10, 1.99), Mixed | White (48.3%), Black (38.6%), Hispanic (11.9%), Asian (7.5%), Other (2.8%) | Cognitive reappraisal, suppression, self-blame, acceptance, rumination, positive refocusing, refocus on planning, positive reappraisal, putting into perspective, others-blame & catastrophizing (CERQ) | Physical, psychological, sexual IPV (CTS2) |
| Zhou et al., 2023 | 530, Community, China | (20.66, 1.64) [18-32], Mixed |  | Difficulties in ER* (DERS)  Suppression and Cognitive Appraisal (ERQ) | Aggression total score (BPAQ) |
| Xie et al., 2023 | 492,  Offenders, China | (34 06, 6 6)  [18-47], Male |  | Suppression and Cognitive Appraisal (ERQ) | Impulsive & Premeditated aggression (IPAS) |
| Hammett et al., 2024 | 215,  Students, US | (20, 2.3)  [18-37], Mixed | White (77.2%), Black/African American (5.6%), Asian (3.3%), American Indian/Alaska Native (0.9%), 0.5%  Native Hawaiian or other Pacific Islander (0.5%) Multiracial (8.4%),  and Other (3.3%) | Difficulties in ER* (DERS) | Physical & psychological, IPV (CTS2) |
| Quan et al., 2024 | 1006, Students, China | (19.63 0.82), Mixed | Chinese | Anger rumination (ARS) | Aggression total score (BPAQ) |
| Bates et al., 2024 | 225, Community, UK | (30.68, 12.20)  [18-77], Mixed |  | Difficulties in ER* (DERS) | Physical, Verbal, Hostility, Anger (BPAQ) |
| Kersten & Greitemeyer, 2024 | 403, Community, Austria | (27.05, 8.44), Mixed |  | Anger rumination (ARS) | Aggression (BPAQ-SF) |
| Kyranides et al., 2024 | 306, Community, UK | (33.79, 13.29) [19-81], Mixed |  | Self-blame, rumination, others-blame & catastrophizing (CERQ) | Physical, Verbal, Hostility, Anger (BPAQ) |
| Hatfield et al., 2023 | 601, Community, US | (41.60, 12.11)  [18-65], Mixed | Black (96.7%) | Non-acceptance,  Goal-direct behaviour, Control difficulties,  Emotional awareness, Emotional clarity, Emotion regulation strategies (DERS) | Aggression (BQ-S) |
| Douadi et al., 2023 | 1845, Offenders, Canada | (37.29, 11.15) [18-88],  Male |  | Affect regulation (IASC) | Physical, psychological, sexual IPV (CTS2) |
| Hou et al., 2024 | 1186, Students, China | (20.78, 1.86) [16-30], Mixed |  | Anger rumination (DAQ) | Proactive & Reactive (RPQ) |
| Larsson et al., 2023 | 538, Community, Sweden | (39.78, 12.28), Mixed |  | Anger rumination (ARS)  Suppression and Cognitive Appraisal (ERQ) | Physical, Verbal, Hostility, Anger (BPAQ) |
| Kavakli et al., 2024 | 325, Students, Turkey | (21.20, 3.09) [18-50],  Mixed |  | Difficulties in ER* (DERS) | Aggression total score (BPAQ) |
| Hayes et al., 2024 | 715, Students, US | (19.37, 1.42), Mixed | White (78.7%) | Emotion dysregulation (ADI) | Reactive & Proactive (PCS-20) |
|  | 780, Students, US | (19.42, 1.44), Mixed | White (75.3%) | Emotion dysregulation (ADI) | Reactive & Proactive (PCS-20) |
| Edwards et al., 2023 | 230, Community, US | (19.84, 1.65) [18-24], Mixed | American Indian/Alaska native (30.4%), Multiracial (69.6%) | Difficulties in ER* (DERS) | Physical, psychological, sexual IPV (SGM-CTS2) |
| Ontiveros et al., 2023 | 13,  Offenders, US | (30.23, 8.99), Male | Hispanic/Latino (92.3%) Native American (7.7%) | Difficulties in ER* (DERS)  Control Difficulties (DERS) | Physical IPV (CTS-2) |
|  | 47, Offenders, US | (31.30, 8.99), Male | Hispanic (93.6%), African America (6.4%) | Difficulties in ER* (DERS)  Control Difficulties (DERS) | Physical IPV (CTS-2) |
| García-Sancho et al., 2015 | 243, Students, Spain | (21.78, 4.38) [19-54], Mixed |  | Anger rumination (DAQ) | Physical, Verbal (BPAQ) |
| Pu et al., 2022 | 203, Community, US | (26.1, 5.87), Female | White (50.7%), African American (46.8%), Asian (1%), Native American (1.5%), Hispanic (3%), Biracial (5.5%) | Emotion regulation (NMRS) | IPV perpetration (CTS-2S) |
|  | 151, Community, US | (28.9, 6.10), Male | White (54%), African America (45.3%), Asian (7%), Hispanic (4%), Biracial (4.7%) | Emotion regulation (NMRS) | IPV perpetration (CTS-2S) |
| Sani et al., 2017 | 117, Community, Iran | (36.59, 9.39) [24-62], Mixed |  | Non-acceptance,  Goal-direct behaviour, Control difficulties,  Emotional awareness, Emotional clarity, Emotion regulation strategies (DERS) | Aggression (AAI) |
| Landa et al., 2024 | 462,  Students, Spain | (20.79, 1.98), Male |  | Suppression (MARS) | Dating Violence (DVQ-R) |
| Clear et al., 2020 | 383,  Students, Australia | (19.6, 1.58) [16-23], Mixed | White (70%), Asian (19%), Australian First People or Pacific Islander (2%), Other (9%) | Anger rumination and suppression (SAIR) | Aggression (YSR) |
| Ciesinski et al., 2022 | 105,  Students,  US | (21.13, 5.76) [18-61], Mixed | White (52.4%), African American (23.8%), or Asian (11.4%); non-Hispanic/Latinx (69.5%) | Non-acceptance,  Goal-direct behaviour, Control difficulties,  Emotional awareness, Emotional clarity, Emotion regulation strategies (DERS)  Anger Rumination (SAIR & RRS) | Physical, Verbal, Hostility, Anger (BPAQ) |
| Zheng et al., 2024 | 491,  Community, US | (30.52, 8.85), Male | White (63.54%), African American or Black (11.), Hispanic or Latino (11%), Asian American or Pacific Islander (8.76%) | Difficulties in ER* (DERS) | Sexual aggression (SES) |
| Chung et al., 2016 | 306,  Offenders,  China | Violent  (36.76, 10.51), Male  Control (36.32, 10.23), Male |  | Suppression (CECS) | Group difference |
| Stefanile et al., 2021 | 266,  Offenders,  Italy | Violent  (43.4, 12.6), Male  Control  (40.5, 15.25), Male |  | Non-acceptance,  Goal-direct behaviour, Control difficulties,  Emotional awareness, Emotional clarity, Emotion regulation strategies (DERS) | Group difference |

*Note*: DERS = difficulties in emotion regulation scale, Difficulties in ER* (DERS) = total score of DERS subscales, ARS = anger rumination scale, ERQ = emotion regulation questionnaire, RRQ = rumination and reflection scale, CERQ = cognitive emotion regulation questionnaire, DRS = dissipation-rumination scale, AAQ-11 = acceptance and action questionnaire-11, RSQ = response styles questionnaire, TAR = trait anger rumination, RTSQ = rumination thought style questionnaire, TMMS = trait meta-mood scale, ASQ = affective style questionnaire, SAIR = sadness and anger rumination inventory, BPAQ = Buss-Parry aggression questionnaire, CTS2 = conflict tactics scale-2, RPQ = reactive-proactive aggression questionnaire, BPAQ-SF = Buss-Perry aggression questionnaire-short form, APBQ = aggression prosocial behaviour, LHAS-S-A = life history of aggression scale, IPAS = impulsive aggression subscale of the impulsive-premediated aggression scale, SES = revised sexual experiences scales, IPV-GMB = intimate partner violence – gay bisexual men aggression scale, AES = aggression-ES scale, SRASMB = self-report of aggression and social behaviour, RVS = relationship violence scale, SES-SF = the sexual experiences survey—short-form perpetration, AIRS = abuse within intimate relationship scales, DRVQ = dating relationship violence questionnaire, CBS-R = controlling behaviour scale, MMEA-SF = multidimensional measure of emotional abuse-short form, FFAM = form-function aggression measure, VSSI = violence scale and severity index, BDH = Buss-Durkee hostility inventory, BWAQ = aggression questionnaire, BDHI-SF = Chinese version of short-form Buss-Durkee hostility inventory, SRBSR = self-report of behaviour scale-revised, ASR = adult self-report Chinese version, MCVI = MacArthur community violence instrument, SD = the safe dates – physical and psychological violence preparation scales, VAS = attitudes towards violence scale, NMRS = negative mood regulation scale, BQ-S = behaviour-questionnaire-short, EVA = engagement in violence and aggression, PCS = peer conflict scale, IASC = inventory of altered self-capacities, DAS = displaced aggression scale, ADI = abbreviated dysregulation inventory, PCS-20 = peer conflict scale-20, SGM-CTS2 = sexual and gender minatory conflict tactics scale, DAQ = displaced aggression scale, AAI = Ahwaz aggression inventory, SARI = sadness and anger rumination inventory, YSR = youth self-report scale, CECS = Courtauld emotional control scale, CTS-2S = conflict tactics scale-short form, Mixed = female and male sample.

| **Table S2**  Full list of emotion regulation measures, coding and frequency | | | | |
| --- | --- | --- | --- | --- |
| Measure | Abbreviation | Coding | Author/s | Frequency |
| Difficulties in Emotion Regulation Scale | DERS | Difficulty | Gratz & Roemer (2004) | 92 |
| Anger Rumination Scale | ARS | Maladaptive | Sukhodolsky et al (2001) | 34 |
| Emotion Regulation Questionnaire | ERQ | Adaptive & Maladaptive | Gross & John (2003) | 12 |
| Rumination and Reflection Questionnaire | RRQ | Maladaptive | Trapnell & Campbell (1999) | 7 |
| Cognitive Emotion Regulation Questionnaire | CERQ | Adaptive & Maladaptive | Garnefski, Kraaij & Spinhoven (2001) | 8 |
| Ruminative Responses Scale | RRS | Maladaptive | Nolen-Hoeksema & Morrow (1991) | 6 |
| Dissipation-Rumination Scale | DRS | Maladaptive | Capara (1986) | 5 |
| Sadness and Anger Rumination Inventory | SAIR | Adaptive | Peled & Moretti. (2007) | 3 |
| Negative Mood Regulation Scale | NMRS | Difficulty | Catanzaro & Mearns (1990) | 3 |
| Acceptance and Action Questionnaire-11 | AAQ-11 | Adaptive & Maladaptive | Bond et al. (2011) | 2 |
| Displaced Aggression Scale | DAQ | Maladaptive | Denson  et al. (2006) | 2 |
| Trait Meta-Mood Scale | TMMS | Difficulty | Salovey et al. (1995) | 2 |
| Responses Styles Questionnaire | RSQ | Maladaptive | Treynor et al. (2003) | 2 |
| The Abbreviated Dysregulation Inventory | ADI | Difficulty | Mezzich  et al. (2001) | 2 |
| Trait Anger Rumination | TAR | Maladaptive | Sukhodolsky et al. (2001) | 1 |
| Acceptance and Action Questionnaire | AAQ | Maladaptive | Hayes et al. (2004) | 1 |
| Rumination Thought Style Questionnaire | RTSQ | Maladaptive | Brinker & Dozois (2009) | 1 |
| Affective Style Questionnaire | ASQ | Adaptive | Hofman & Kashdan (2010) | 1 |
| Inventory of Altered Self-Capacities | IASC | Difficulty | Briere (2000) | 1 |
| Measurement of Affect Regulation Styles | MARS | Maladaptive | Larsen  & Prizmic (2004) | 1 |
| The Courtauld Emotional Control Scale | CECS | Maladaptive | Watson & Greer (1983) | 1 |

| **Table S3**  Full list of aggression measures | | | | |
| --- | --- | --- | --- | --- |
| Measure | Abbreviation | Coding | Author/s | Frequency |
| Buss-Perry Aggression Questionnaire | BPAQ | Physical, Sexual& Other | Buss & Perry (1992) | 63 |
| Conflict Tactics Scale-2 | CTS2 | Physical, Sexual & Other | Straus et al. (1996) | 39 |
| Reactive-Proactive Aggression Questionnaire | RPQ | Other | Raine et al. (2006) | 15 |
| Impulsive Aggression Subscale of the Impulsive-Premeditated Aggression Scale | IPAS | Other | Stanford et al. (2003) | 5 |
| Life History of Aggression Scale | LHAS-S-A | Other | Coccaro et al. (1995) | 3 |
| Revised Sexual Experiences Scales | SES | Sexual | Koss et al. (1987); Parkhill & Abbey (2008) | 3 |
| Buss-Perry Aggression Questionnaire-Short form | BPAQ-SF | Physical & Other | Bryant & Smith (2001) | 2 |
| Aggression Prosocial Behaviour Questionnaire | APBQ | Physical & Other | Boxer et al. (2004) | 2 |
| The Behaviour Questionnaire - Short | BQ-S | Physical | Gillikin et al. (2016) | 2 |
| Peer Conflict Scale-20 | PCS-20 | Other | Marsee & Frick (2007) | 2 |
| Conflict Tactics Scale-2 Short Form | CTS-2S | Physical & Other | Straus & Douglas (2004) | 2 |
| Violence Scale and Severity Index | VSSI | Other | Valdez Santiago et al. (2006) | 2 |
| Attitude Towards Violence Scale | VAS | Physical | Capara et al. (1900) | 2 |
| (The Safe Dates—Physical and Psychological Violence Perpetration scales) | SD | Physical & Psychological | Foshee et al. (1998) | 2 |
| The Sexual Experiences Survey—Short-Form Perpetration | SES-SF | Sexual | Koss et al. (2007) | 2 |
| Abuse Within Intimate Relationships Scales | AIRS | Physical | Borjesson et al. (2003) | 2 |
| Self-report of Aggression and Social Behaviour | SRASMB | Physical | Morales & Crick (1998) | 2 |
| Dating Relationship Violence Questionnaire | DRVQ | Other | Swahn et al. (2008) | 1 |
| Intimate Partner Violence – Gay Bisexual Men | IPV-GBM | Physical, Sexual & Other | Stephenson & Finneran (2013) | 1 |
| Aggression Scale |  | Other | Isobe & Hishinuma (2007) | 1 |
| Explicit Aggression Scale | EAS | Other | Borders et al. (2007) | 1 |
| Aggression-ES scale | AES | Other | Murray et al. (2022) | 1 |
| Relationship violence scale | RVS | Physical | Whitmire et al. (1999) | 1 |
| Controlling behaviour scale | CBS-R | Other | Sleath et al. (2018) | 1 |
| Multidimensional Measure of Emotional Abuse-Short Form | MMEA-SF | Other | Maldonado et al. (2022) | 1 |
| Form-Function Aggression Measure | FFAM | Other | Little et al. (2003) | 1 |
| Buss-Durkee Hostility Inventory | BDHI | Other | Buss & Durkee (1957) | 1 |
| Aggression Questionnaire | BWAQ | Other | Buss & Warren (2000) | 1 |
| Chinese version of Short-form Buss-Durkee Hostility Inventory | BDHIS-SF | Other | Lin et al. (2008) | 1 |
| Self-report of behaviour scale-revised | SRBSR | Other | Roderick et al. (1998); Parrott & Peterson (2008) | 1 |
| Adult self-report – Chinese version | ASR | Other | Achenbach & Rescorla (2003) | 1 |
| MacArthur Community Violence Instrument | MCVI | Other | Monahan & Steadman (1994) | 1 |
| Engagement in Violent and Aggression | EVA | Physical | Caprara et al., (1990) | 1 |
| Peer Conflict Scale | PCS | Other | Marsee et al. (2011) | 1 |
| Sexual and Gender Minority Conflict Tactics Scale | SGM-CTS2 | Physical, Psychological & Sexual | Dyar et al. (2021) | 1 |
| Ahwaz Aggression Inventory | AAI | Other | Zahedifar et al. (2000) | 1 |
| Youth Self-Report Scale | YSR | Other | Achenbach & Rescorla (2001) | 1 |

**Figure S1**

*Results of cumulative meta-analysis, showing effects over time by year of publication (top panel = Aggression ~ Adaptive ER strategies, middle panel = Aggression ~ Maladaptive ER Strategies, bottom panel = Aggression ~ Difficulties in ER)*

**Figure S2**

*Results of p-curve analysis for Aggression ~ Adaptive ER strategies*

**Figure S3**

*Results of p-curve analysis for Aggression ~ Maladaptive ER strategies*

**Figure S4**

*Results of p-curve analysis for Aggression ~ Difficulties in ER*

| Table S4  *AXIS quality assessment tool for included studies in meta-analysis* | | | | | | | | | | | | | | | | | | | | |
| --- | --- | --- | --- | --- | --- | --- | --- | --- | --- | --- | --- | --- | --- | --- | --- | --- | --- | --- | --- | --- |
| Author/s | Q1 | Q2 | Q3 | Q4 | Q5 | Q6 | Q7 | Q8 | Q9 | Q10 | Q11 | Q12 | Q13 | Q14 | Q15 | Q16 | Q17 | Q18 | Q19 | Q20 |
|  |  |  |  |  |  |  |  |  |  |  |  |  |  |  |  |  |  |  |  |  |
| EL-Kim et al., 2022 | Y | Y | N | Y | Y | Y | N | Y | Y | Y | Y | Y | DK | N | Y | Y | Y | Y | N | Y |
| Mellat et al., 2023 | Y | Y | Y | Y | Y | Y | N | Y | Y | Y | Y | Y | DK | N | Y | Y | Y | Y | N | Y |
| Anestis et al., 2019 | Y | Y | N | Y | Y | Y | N | Y | Y | Y | Y | Y | DK | N | DK | Y | Y | Y | N | Y |
| Barlett, 2022 | Y | Y | Y | Y | Y | Y | N | Y | Y | Y | Y | Y | DK | N | DK | Y | Y | Y | N | Y |
| Bell et al., 2022 | Y | Y | N | Y | Y | Y | N | Y | Y | Y | Y | Y | DK | N | Y | Y | Y | Y | N | Y |
| Bilton et al., 2016 | Y | Y | N | Y | Y | Y | N | Y | Y | Y | Y | Y | DK | N | Y | Y | Y | Y | N | Y |
| Borders et al., 2007 | Y | Y | N | Y | Y | Y | N | Y | Y | Y | Y | Y | DK | N | N | Y | Y | Y | DK | Y |
| Borders et al., 2010 | Y | Y | N | Y | Y | Y | N | Y | Y | Y | Y | Y | DK | N | Y | Y | Y | Y | DK | Y |
| Borders et al., 2011 | Y | Y | N | Y | Y | Y | N | Y | Y | Y | Y | Y | DK | N | DK | Y | Y | Y | DK | Y |
| Buyuks et al., 2023 | Y | Y | N | Y | Y | Y | N | Y | Y | Y | Y | Y | DK | N | Y | Y | Y | Y | N | Y |
| Dilek et al., 2021 | Y | Y | N | Y | Y | Y | N | Y | Y | Y | Y | Y | DK | N | Y | Y | Y | Y | N | Y |
| Chatzimike-Levidi & Collard, 2023 | Y | Y | N | Y | Y | Y | N | Y | Y | Y | Y | Y | N | N | Y | Y | Y | Y | N | Y |
| Hoover et al., 2021 | Y | Y | Y | Y | Y | Y | N | Y | Y | Y | Y | Y | Y | Y | Y | Y | Y | Y | N | Y |
| Demichelis et al., 2022 | Y | Y | Y | Y | Y | Y | N | Y | Y | Y | Y | Y | N | N | Y | Y | Y | Y | N | Y |
| Donahue et al., 2014 | Y | Y | N | Y | Y | Y | Y | Y | Y | Y | Y | Y | N | N | Y | Y | Y | Y | DK | Y |
| Edwards et al., 2017 | Y | Y | Y | Y | Y | Y | N | Y | Y | Y | Y | Y | DK | N | N | Y | Y | Y | DK | Y |
| Fresnics et al., 2017 | Y | Y | N | Y | Y | Y | N | Y | Y | Y | Y | Y | DK | N | DK | Y | Y | Y | N | Y |
| Garofalo et al., 2020 | Y | Y | N | Y | Y | Y | N | Y | Y | Y | Y | Y | DK | N | Y | Y | Y | Y | DK | Y |
| Garofalo et al., 2016 | Y | Y | N | Y | Y | Y | N | Y | Y | Y | Y | Y | DK | N | Y | Y | Y | Y | N | Y |
| Garofalo et al., 2021 | Y | Y | N | Y | Y | Y | N | Y | Y | Y | Y | Y | DK | N | Y | Y | Y | Y | N | Y |
| Garofalo et al., 2018 | Y | Y | N | Y | Y | Y | N | Y | Y | Y | Y | Y | DK | N | Y | Y | Y | Y | DK | Y |
| Garofalo et al., 2017 | Y | Y | N | Y | Y | Y | N | Y | Y | Y | Y | Y | DK | N | Y | Y | Y | Y | DK | Y |
| Gómez-Leal et al., 2022 | Y | Y | Y | Y | Y | Y | N | Y | Y | Y | Y | Y | Y | N | Y | Y | Y | Y | N | Y |
| Grigorian et al., 2022 | Y | Y | Y | Y | Y | Y | N | Y | Y | Y | Y | Y | DK | N | DK | Y | Y | Y | DK | Y |
| Grigorian et al., 2019 | Y | Y | N | Y | Y | Y | N | Y | Y | Y | Y | Y | DK | N | N | Y | Y | Y | DK | Y |
| Guerra et al., 2017 | Y | Y | N | Y | Y | Y | N | Y | Y | Y | Y | Y | N | N | N | Y | Y | Y | N | Y |
| Guzmán-González et al., 2016 | Y | Y | N | Y | Y | Y | N | Y | Y | Y | Y | Y | N | N | Y | Y | Y | Y | N | Y |
| Hasegawa et al., 2022a | Y | Y | Y | Y | Y | Y | N | Y | Y | Y | Y | Y | N | N | Y | Y | Y | Y | N | Y |
| Hayes et al., 2021 | Y | Y | Y | Y | Y | Y | N | Y | Y | Y | Y | Y | N | N | Y | Y | Y | Y | N | Y |
| He et al., 2022 | Y | Y | N | Y | Y | Y | N | Y | Y | Y | Y | Y | Y | N | DK | Y | Y | Y | N | Y |
| Kirwan et al., 2019a | Y | Y | Y | Y | Y | Y | N | Y | Y | Y | Y | Y | N | N | N | Y | Y | Y | N | DK |
| Kirwan et al., 2019b | Y | Y | Y | Y | Y | Y | N | Y | Y | Y | Y | Y | N | N | N | Y | Y | Y | DK | Y |
| Lee et al., 2019 | Y | Y | N | Y | Y | Y | N | Y | Y | Y | Y | Y | DK | N | Y | Y | Y | Y | DK | DK |
| Lewis et al., 2014 | Y | Y | N | Y | Y | Y | N | Y | Y | Y | Y | Y | N | Y | Y | Y | Y | Y | N | DK |
| Li et al., 2022 | Y | Y | N | Y | Y | Y | N | Y | Y | Y | Y | Y | Y | N | Y | Y | Y | Y | N | Y |
| Lilly et al., 2014 | Y | Y | N | Y | Y | Y | N | Y | Y | Y | Y | Y | DK | N | N | Y | Y | Y | DK | Y |
| Logoz et al., 2023 | Y | Y | N | Y | Y | Y | DK | Y | Y | Y | Y | Y | Y | N | Y | Y | Y | Y | N | Y |
| Long et al., 2014 | Y | Y | N | Y | Y | Y | N | Y | Y | Y | Y | Y | DK | N | Y | Y | Y | Y | DK | DK |
| Mancke et al., 2017 | Y | Y | N | Y | Y | Y | N | Y | Y | Y | Y | Y | N | N | N | Y | Y | Y | DK | Y |
| Mansfield et al., 2019 | Y | Y | N | Y | Y | Y | N | Y | Y | Y | Y | Y | DK | N | Y | Y | Y | Y | DK | Y |
| Martino et al., 2015 | Y | Y | N | Y | Y | Y | N | Y | Y | Y | Y | Y | N | N | N | Y | Y | Y | N | Y |
| Martino et al., 2018 | Y | Y | N | Y | Y | Y | N | Y | Y | Y | Y | Y | N | N | DK | Y | Y | Y | DK | Y |
| Massa et al., 2019 | Y | Y | N | Y | Y | Y | N | Y | Y | Y | Y | Y | N | N | Y | Y | Y | Y | DK | Y |
| Miles et al., 2016 | Y | Y | N | Y | Y | Y | N | Y | Y | Y | Y | Y | Y | N | Y | Y | Y | Y | N | Y |
| Oliveros et al., 2021 | Y | Y | N | Y | Y | Y | N | Y | Y | Y | Y | Y | DK | N | Y | Y | Y | Y | N | Y |
| Ortiz et al., 2015 | Y | Y | N | Y | Y | Y | N | Y | Y | Y | Y | Y | DK | N | Y | Y | Y | Y | DK | Y |
| Peters et al., 2015 | Y | Y | N | Y | Y | Y | N | Y | Y | Y | Y | Y | DK | N | Y | Y | Y | Y | DK | Y |
| Pickett et al., 2017 | Y | Y | N | Y | Y | Y | N | Y | Y | Y | Y | Y | DK | N | N | Y | Y | Y | N | DK |
| Pollard et al., 2021 | Y | Y | N | Y | Y | Y | N | Y | Y | Y | Y | Y | DK | N | Y | Y | Y | Y | N | Y |
| Preston et al., 2020 | Y | Y | Y | Y | Y | Y | N | Y | Y | Y | Y | Y | DK | N | Y | Y | Y | Y | N | Y |
| Price et al., 2014 | Y | Y | N | Y | Y | Y | N | Y | Y | Y | Y | Y | DK | N | N | Y | Y | Y | DK | DK |
| Pugliese et al., 2015 | Y | Y | Y | Y | Y | Y | N | Y | Y | Y | Y | Y | N | N | DK | Y | Y | Y | N | Y |
| Puhalla et al., 2020 | Y | Y | N | Y | Y | Y | N | Y | Y | Y | Y | Y | DK | N | N | Y | Y | Y | N | Y |
| Quan et al., 2020 | Y | Y | N | Y | Y | Y | N | Y | Y | Y | Y | Y | DK | N | Y | Y | Y | Y | N | Y |
| Quan et al., 2021 | Y | Y | Y | Y | Y | Y | N | Y | Y | Y | Y | Y | N | N | Y | Y | Y | Y | N | Y |
| Quan et al., 2019 | Y | Y | N | Y | Y | Y | N | Y | Y | Y | Y | Y | Y | N | Y | Y | Y | Y | N | Y |
| Sanchez-Ruiz et al., 2018 | Y | Y | N | Y | Y | Y | N | Y | Y | Y | Y | Y | DK | N | DK | Y | Y | Y | DK | Y |
| Scott et al., 2014 | Y | Y | Y | Y | Y | Y | N | Y | Y | Y | Y | Y | DK | N | Y | Y | Y | Y | DK | Y |
| Shamsipour et al., 2018 | Y | Y | N | Y | Y | Y | N | Y | Y | Y | Y | Y | N | N | N | Y | Y | Y | N | Y |
| Shorey et al., 2015 | Y | Y | N | Y | Y | Y | N | Y | Y | Y | Y | Y | Y | N | DK | Y | Y | Y | N | Y |
| Shorey et al., 2011a | Y | Y | N | Y | Y | Y | N | Y | Y | Y | Y | Y | DK | N | Y | Y | Y | Y | DK | Y |
| Shorey et al., 2011b | Y | Y | N | Y | Y | Y | N | Y | Y | Y | Y | Y | N | N | N | Y | Y | Y | DK | Y |
| Shorey et al., 2014 | Y | Y | N | Y | Y | Y | N | Y | Y | Y | Y | Y | DK | N | DK | Y | Y | Y | DK | Y |
| Sotelo et al., 2013 | Y | Y | N | Y | Y | Y | N | Y | Y | Y | Y | Y | DK | N | DK | Y | Y | Y | DK | DK |
| Stappenbeck et al., 2016 | Y | Y | N | Y | Y | Y | N | Y | Y | Y | Y | Y | DK | N | DK | Y | Y | Y | DK | DK |
| Terzi et al., 2017 | Y | Y | N | Y | Y | Y | N | Y | Y | Y | Y | Y | DK | N | DK | N | Y | Y | N | Y |
| Thiessen et al., 2018 | Y | Y | N | Y | Y | Y | N | Y | Y | Y | Y | Y | DK | N | DK | Y | Y | Y | N | Y |
| Trombetta et al., 2023 | Y | Y | N | Y | Y | Y | N | Y | Y | Y | Y | Y | DK | N | Y | Y | Y | Y | N | Y |
| Turner et al., 2015 | Y | Y | N | Y | Y | Y | N | Y | Y | Y | Y | Y | DK | N | DK | Y | Y | Y | DK | Y |
| Velotti et al., 2016 | Y | Y | N | Y | Y | Y | N | Y | Y | Y | Y | Y | DK | N | Y | Y | Y | Y | N | Y |
| Wahlstrom et al., 2015 | Y | Y | N | Y | Y | Y | N | Y | Y | Y | Y | Y | DK | N | Y | Y | Y | Y | N | Y |
| Wang et al., 2018 | Y | Y | N | Y | Y | Y | N | Y | Y | Y | Y | Y | DK | N | DK | Y | Y | Y | DK | Y |
| Wang et al., 2020 | Y | Y | N | Y | Y | Y | N | Y | Y | Y | Y | Y | Y | N | DK | Y | Y | Y | N | Y |
| Watkins et al., 2016 | Y | Y | N | Y | Y | Y | N | Y | Y | Y | N | Y | DK | N | N | Y | Y | Y | DK | Y |
| Wei et a., 2020 | Y | Y | N | Y | Y | Y | N | Y | Y | Y | Y | Y | Y | N | Y | Y | Y | Y | N | Y |
| White et al., 2014 | Y | Y | N | Y | Y | Y | N | Y | Y | Y | Y | Y | DK | N | DK | Y | Y | Y | DK | Y |
| Yang, 2020 | Y | Y | N | Y | Y | Y | Y | Y | Y | Y | Y | Y | N | Y | Y | Y | Y | Y | N | Y |
| Garofalo et al., 2017 | Y | Y | N | Y | Y | Y | N | Y | Y | Y | Y | Y | N | N | Y | Y | Y | Y | DK | Y |
| Cen et al., 2022 | Y | Y | N | Y | Y | Y | Y | Y | Y | Y | Y | Y | N | Y | Y | Y | Y | Y | N | Y |
| Chen et al., 2020 | Y | Y | N | Y | Y | Y | N | Y | Y | Y | Y | Y | Y | N | Y | Y | Y | Y | N | Y |
| Velotti et al., 2017 | Y | Y | N | Y | Y | Y | N | Y | Y | Y | Y | Y | DK | N | Y | Y | Y | Y | DK | Y |
| Espirito-Santo et al., 2022 | Y | Y | N | Y | Y | Y | N | Y | Y | Y | Y | Y | DK | N | Y | Y | Y | Y | N | Y |
| Avnaim et al., 2022 | Y | Y | N | Y | Y | Y | N | Y | Y | Y | Y | Y | N | N | Y | Y | Y | Y | DK | Y |
| Peled et al., 2010 | Y | Y | N | Y | Y | Y | N | Y | Y | Y | Y | Y | DK | N | Y | Y | Y | Y | DK | Y |
| Orozco-Vargas et al., 2021 | Y | Y | N | Y | Y | Y | N | Y | Y | Y | Y | Y | N | N | Y | Y | Y | Y | N | Y |
| Tull et al., 2007 | Y | Y | N | Y | Y | Y | N | Y | Y | Y | Y | Y | DK | N | Y | Y | Y | Y | DK | Y |
| Fern et al., 2023 | Y | Y | N | Y | Y | Y | N | Y | Y | Y | Y | Y | Y | N | DK | Y | Y | Y | N | Y |
| Han et al., 2020 | Y | Y | N | Y | Y | Y | N | Y | Y | Y | Y | Y | Y | N | Y | Y | Y | Y | DK | Y |
| Hasegawa et al., 2022b | Y | Y | N | Y | Y | Y | N | Y | Y | Y | Y | Y | Y | N | Y | Y | Y | Y | N | Y |
| Hosie et al., 2021 | Y | Y | Y | Y | Y | Y | N | Y | Y | Y | Y | Y | N | N | Y | Y | Y | Y | DK | Y |
| Hosie et al., 2022 | Y | Y | N | Y | Y | Y | N | Y | Y | Y | Y | Y | Y | N | Y | Y | Y | Y | N | Y |
| Maxwell et al., 2007 | Y | Y | N | Y | Y | Y | N | Y | Y | Y | Y | Y | DK | N | DK | Y | Y | Y | N | Y |
| Rogier et al., 2019 | Y | Y | N | Y | Y | Y | N | Y | Y | Y | Y | Y | DK | N | Y | Y | Y | Y | DK | Y |
| Lin et al., 2020 | Y | Y | N | Y | Y | Y | N | Y | Y | Y | Y | Y | DK | N | Y | Y | Y | Y | DK | Y |
| Ray et al., 2020 | Y | Y | N | Y | Y | Y | N | Y | Y | Y | Y | Y | Y | N | DK | Y | Y | Y | DK | Y |
| Li et al., 2019 | Y | Y | N | Y | Y | Y | N | Y | Y | Y | Y | Y | Y | N | Y | Y | Y | Y | DK | DK |
| Caprara et al., 2014 | Y | Y | N | Y | Y | Y | N | Y | Y | Y | DK | Y | Y | N | DK | Y | Y | Y | DK | Y |
| Sarrate-Costa et al., 2023 | Y | Y | N | Y | Y | Y | N | Y | Y | Y | Y | Y | N | N | Y | Y | Y | Y | DK | Y |
| Ireland et al., 2020 | Y | Y | N | Y | Y | Y | N | Y | Y | Y | Y | Y | DK | N | Y | Y | Y | Y | N | Y |
| Holley et al., 2017 | Y | Y | N | Y | Y | Y | N | Y | Y | Y | Y | Y | N | N | DK | Y | Y | Y | N | Y |
| Conzemius et al., 2021 | Y | Y | N | Y | Y | Y | N | Y | Y | Y | Y | Y | DK | N | N | Y | Y | Y | N | Y |
| Velotti et al., 2020 | Y | Y | N | Y | Y | Y | N | Y | Y | Y | Y | Y | DK | N | Y | Y | Y | Y | N | Y |
| Kayha et al., 2019 | Y | Y | N | Y | Y | Y | N | Y | Y | Y | Y | Y | Y | N | DK | Y | Y | Y | N | Y |
| Marín-Morales et al., 2020 | Y | Y | Y | Y | Y | Y | Y | Y | Y | Y | Y | Y | DK | N | Y | Y | Y | Y | N | Y |
| *Note:* Q = question on AXIS, Y = yes, N = no, DK = don’t know | | | | | | | | | | | | | | | | | | | | |

**References for Studies Included in Meta-analysis (Table S1)**

Anestis, M. D., Anestis, J. C., Selby, E. A., & Joiner, T. E. (2009). Anger rumination across forms of aggression. *Personality and Individual Differences*, *46*(2), 192-196. https://doi.org/10.1016/j.paid.2008.09.026

Avnaim, S., Murphy, C. M., & Miles-McLean, H. A. (2022). How Do Women in Treatment for Intimate Partner Violence Perpetration Perceive Their Abusive Behavior? *Psychology of Violence*, *12*(5), 324-332. https://doi.org/10.1037/vio0000427

Barlett, C. P. (2022). Thinking through situations: The mediating role of rumination in the relationship between need for cognition and aggression. *Aggressive Behavior*. https://doi.org/10.1002/ab.22068

Bell, K. M., Howard, L., & Cornelius, T. L. (2022). Emotion dysregulation as a moderator of the association between relationship dependency and female-perpetrated dating aggression. *Journal of Interpersonal Violence*, *37*(5), NP2891-NP2911. https://doi.org/10.1177/0886260520945678

Bliton, C. F., Wolford-Clevenger, C., Zapor, H., Elmquist, J. A., Brem, M. J., Shorey, R. C., & Stuart, G. L. (2016). Emotion Dysregulation, Gender, and Intimate Partner Violence Perpetration: An Exploratory Study in College Students. *Journal of Family Violence*, *31*(3), 371-377. https://doi.org/10.1007/s10896-015-9772-0

Borders, A., Barnwell, S. S., & Earleywine, M. (2007). Alcohol-aggression expectancies and dispositional rumination moderate the effect of alcohol consumption on alcohol-related aggression and hostility. *Aggressive Behavior*, *33*(4), 327-338. https://doi.org/10.1002/ab.20187

Borders, A., Earleywine, M., & Jajodia, A. (2010). Could mindfulness decrease anger, hostility, and aggression by decreasing rumination? *Aggressive Behavior*, *36*(1), 28-44. https://doi.org/10.1002/ab.20327

Borders, A., & Liang, C. T. H. (2011). Rumination partially mediates the associations between perceived ethnic discrimination, emotional distress, and aggression. *Cultural Diversity and Ethnic Minority Psychology*, *17*(2), 125-133. https://doi.org/10.1037/a0023357

Buyuks, alyaci Tunc, E., & Gul, O. (2023). Difficulties in Emotion Regulation: Are They the Preventable Cause of Suicide, Impulsivity, and Aggression in Schizophrenia and Bipolar Disorder? *Psychiatr Danub*, *35*(1), 38-46. https://doi.org/10.24869/psyd.2023.38

Caprara, G. V., Tisak, M. S., Aless, ri, G., Fontaine, R. G., Fida, R., & Paciello, M. (2014). The contribution of moral disengagement in mediating individual tendencies toward aggression and violence. *Developmental Psychology*, *50*(1), 71-85. https://doi.org/10.1037/a0034488

Celik, D., Alpay, E. H., Celebi, B., & Turkali, A. (2021). Intolerance of uncertainty, rumination, post-traumatic stress symptoms and aggression during COVID-19: A serial mediation model. *European Journal of Psychotraumatology*, *12*(1). https://doi.org/10.1080/20008198.2021.1953790

Cen, Y., Su, S., Dong, Y., Xia, L. X., & Xia, L.-X. (2022). Longitudinal effect of self-control on reactive-proactive aggression: Mediating roles of hostile rumination and moral disengagement. *Aggressive Behavior*, *48*(6), 583-594. https://doi.org/10.1002/ab.22046

Chatzimike-Levidi, M. D., & Collard, J. J. (2022). An integrated model of aggression: Links between core self-evaluations, anger rumination and forgiveness. *Current Psychology: A Journal for Diverse Perspectives on Diverse Psychological Issues*. https://doi.org/10.1007/s12144-022-04077-9

Chen, J., Zhang, C., Wang, Y., & Xu, W. (2020). A longitudinal study of inferiority impacting on aggression among college students: The mediation role of cognitive reappraisal and expression suppression. *Personality and Individual Differences*, *157*. https://doi.org/10.1016/j.paid.2020.109839

Conzemius, D. J., Brem, M. J., Wettersten, K. B., & Stuart, G. L. (2021). Traditional gender roles and emotion dysregulation as risk factors for intimate partner violence perpetration among college men. *Partner Abuse*, *12*(3), 343-360. https://doi.org/10.1891/PA-2020-0033

Demichelis, O. P., Grainger, S. A., Burr, L., & Henry, J. D. (2023). Emotion regulation mediates the effects of sleep on stress and aggression. *Journal of Sleep Research*, *32*(3), 1-9. https://doi.org/10.1111/jsr.13787

Donahue, J. J., Goranson, A. C., McClure, K. S., & Van Male, L. M. (2014). Emotion dysregulation, negative affect, and aggression: A moderated, multiple mediator analysis. *Personality and Individual Differences*, *70*, 23-28. https://doi.org/10.1016/j.paid.2014.06.009

Edwards, E. R., & Wupperman, P. (2017). Emotion regulation mediates effects of alexithymia and emotion differentiation on impulsive aggressive behavior. *Deviant Behavior*, *38*(10), 1160-1171. https://doi.org/10.1080/01639625.2016.1241066

Espirito-Santo, H., Daniel, F., Lemos, L., Simões-Cunha, L., & Grasina, A. (2022). Alexithymia and aggressiveness in old age: Mediation by impulsivity and emotion dysregulation. *Psychology, Society and Education*, *14*(1), 35-43. https://doi.org/10.21071/PSYE.V14I1.14180

Fernandez, S. J., Daffern, M., Moulding, R., & Nedeljkovic, M. (2023). Exploring predictors of aggressive intrusive thoughts and aggressive scripts: Similarities and differences in phenomenology. *Aggressive Behavior*, *49*(2), 141-153. https://doi.org/10.1002/ab.22061

Fresnics, A., & Borders, A. (2017). Angry rumination mediates the unique associations between self-compassion and anger and aggression. *Mindfulness*, *8*(3), 554-564. https://doi.org/10.1007/s12671-016-0629-2

Garofalo, C., Gillespie, S. M., & Velotti, P. (2020). Emotion regulation mediates relationships between mindfulness facets and aggression dimensions. *Aggressive Behavior*, *46*(1), 60-71. https://doi.org/10.1002/ab.21868

Garofalo, C., Holden, C. J., Zeigler-Hill, V., & Velotti, P. (2016). Understanding the connection between self-esteem and aggression: The mediating role of emotion dysregulation. *Aggressive Behavior*, *42*(1), 3-15. https://doi.org/10.1002/ab.21601

Garofalo, C., Neumann, C. S., & Velotti, P. (2021). Psychopathy and aggression: The role of emotion dysregulation. *Journal of Interpersonal Violence*, *36*(23), NP12640-NP12664. https://doi.org/10.1177/0886260519900946

Garofalo, C., & Velotti, P. (2017). Negative emotionality and aggression in violent offenders: The moderating role of emotion dysregulation. *Journal of Criminal Justice*, *51*, 9-16. https://doi.org/10.1016/j.jcrimjus.2017.05.015

Garofalo, C., Velotti, P., & Zavattini, G. C. (2018). Emotion regulation and aggression: The incremental contribution of alexithymia, impulsivity, and emotion dysregulation facets. *Psychology of Violence*, *8*(4), 470-483. https://doi.org/10.1037/vio0000141

Garofalo, C., Velotti, P., Zavattini, G. C., & Kosson, D. S. (2017). Emotion dysregulation and interpersonal problems: The role of defensiveness. *Personality and Individual Differences*, *119*, 96-105. https://doi.org/10.1016/j.paid.2017.07.007

Gómez-Leal, R., Megías-Robles, A., Gutiérrez-Cobo, M. J., Cabello, R., & Fernández-Berrocal, P. (2022). Personal Risk and Protective Factors Involved in Aggressive Behavior. *Journal of Interpersonal Violence*, *37*(3), NP1489-NP1515. https://doi.org/10.1177/0886260520926322

Grigorian, H. L., Brem, M. J., Garner, A., Florimbio, A. R., Wolford-Clevenger, C., & Stuart, G. L. (2020). Alcohol Use and Problems as a Potential Mediator of the Relationship between Emotion Dysregulation and IPV Perpetration. *Psychol Violence*, *10*(1), 91-99. https://doi.org/10.1037/vio0000237

Grigorian, H. L., Garner, A., Florimbio, A. R., Brem, M. J., Wolford-Clevenger, C., Elmquist, J. M., Shorey, R. C., & Stuart, G. L. (2019). Emotion dysregulation as a correlate of intimate partner violence among women arrested for domestic violence. *Partner Abuse*, *10*(1), 98-113. https://doi.org/10.1891/1946-6560.10.1.98

Guerra, R., & White, B. (2017). Psychopathy and Functions of Aggression in Emerging Adulthood: Moderation by Anger Rumination and Gender. *Journal of Psychopathology & Behavioral Assessment*, *39*(1), 35-45. https://doi.org/10.1007/s10862-016-9563-9

Guzmán-González, M., Lafontaine, M.-F., & Levesque, C. (2016). Romantic attachment and physical intimate partner violence perpetration in a Chilean sample: The mediating role of emotion regulation difficulties. *Violence and Victims*, *31*(5), 854-868. https://doi.org/10.1891/0886-6708.VV-D-14-00114

Han, L., Xiao, M., Jou, M., Hu, L., Sun, R., & Zhou, Z. (2020). The long-term effect of media violence exposure on aggression of youngsters. *Computers in Human Behavior*, *106*. https://doi.org/10.1016/j.chb.2020.106257

Hasegawa, A., Matsumoto, N., Yamashita, Y., Tanaka, K., Kawaguchi, J., & Yamamoto, T. (2022a). Correction to: Response inhibition deficits are positively associated with trait rumination, but attentional inhibition deficits are not: aggressive behaviors and interpersonal stressors as mediators. *Psychol Res*, *86*(7), 2301-2303. https://doi.org/10.1007/s00426-021-01582-7

Hasegawa, A., Oura, S.-i., Yamamoto, T., Kunisato, Y., Matsuda, Y., & Adachi, M. (2022b). Causes and consequences of stress generation: Longitudinal associations of negative events, aggressive behaviors, rumination, and depressive symptoms. *Current Psychology: A Journal for Diverse Perspectives on Diverse Psychological Issues*. https://doi.org/10.1007/s12144-022-02859-9

Hayes, N. L., Lloyd-Richardson, E. E., & Marsee, M. A. (2021). Correlates of Relational and Physical Aggression among Peers and Dating Partners in a College Sample. *Journal of Child and Family Studies*, *30*(7), 1697-1711. https://doi.org/10.1007/s10826-021-01973-9

He, C., Mao, J., Yang, Q., Yuan, J., & Yang, J. (2022). Trait Acceptance Buffers Aggressive Tendency by the Regulation of Anger during Social Exclusion. *International Journal of Environmental Research and Public Health*, *19*(22). https://doi.org/10.3390/ijerph192214666

Holley, S. R., Ewing, S. T., Stiver, J. T., & Bloch, L. (2017). The relationship between emotion regulation, executive functioning, and aggressive behaviors. *Journal of Interpersonal Violence*, *32*(11), 1692-1707. https://doi.org/10.1177/0886260515592619

Hoover, R. C., & Jackson, J. B. (2021). Insecure attachment, emotion dysregulation, and psychological aggression in couples. *Journal of Interpersonal Violence*, *36*(19), NP10908-NP10936. https://doi.org/10.1177/0886260519877939

Hosie, J., Dunne, A. L., Meyer, D., & Daffern, M. (2021). Aggressive script rehearsal in adult offenders: Relationships with emotion regulation difficulties and aggressive behavior. *Aggressive Behavior*. https://doi.org/10.1002/ab.22000

Hosie, J., Simpson, K., Dunne, A., & Daffern, M. (2022). A study of the relationships between rumination, anger rumination, aggressive script rehearsal, and aggressive behavior in a sample of incarcerated adult males. *Journal of Clinical Psychology*, *78*(9), 1925-1939. https://doi.org/10.1002/jclp.23341

Ireland, L., J., Lewis, M., Irel, A., C., Derefaka, G., Taylor, L., McBoyle, J., Smillie, L., Chu, S., & Archer, J. (2020). Self-reported psychopathy and aggression motivation: a role for emotions? *Journal of Forensic Psychiatry and Psychology*, *31*(1), 156-181. https://doi.org/10.1080/14789949.2019.1705376

Kayha, Y., & Taskale, N. (2019). Difficulties in emotion regulation, separation anxiety, and impulsivity as predictors of women’s intimate partner violence experiences. *Düşünen Adam: Journal of Psychiatry and Neurological Sciences*, *32*(2), 101-112. [10.14744/dajpns.2019.00016](http://doi.org/10.14744/dajpns.2019.00016)

Kim, E. L., Gentile, D. A., Anderson, C. A., & Barlett, C. P. (2022). Are mindful people less aggressive? The role of emotion regulation in the relations between mindfulness and aggression. *Aggressive Behavior*. https://doi.org/10.1002/ab.22036

Kirwan, M., Lanni, D. J., Warnke, A., Pickett, S. M., & Parkhill, M. R. (2019a). Emotion regulation moderates the relationship between alcohol consumption and the perpetration of sexual aggression. *Violence Against Women*, *25*(9), 1053-1073. https://doi.org/10.1177/1077801218808396

Kirwan, M., Svenson, D. W., Pickett, S. M., & Parkhill, M. R. (2019b). Emotion regulation as a mediator between sleep quality and interpersonal aggression. *Personality and Individual Differences*, *148*, 32-37. https://doi.org/10.1016/j.paid.2019.05.018

Lee, K. D. M., Rodriguez, L. M., Edwards, K. M., & Neal, A. M. (2019). Emotional Dysregulation and Intimate Partner Violence: A Dyadic Perspective. *Psychology of Violence*. https://doi.org/10.1037/vio0000248

Lewis, R. J., Milletich, R. J., Derlega, V. J., & Padilla, M. A. (2014). Sexual minority stressors and psychological aggression in lesbian women’s intimate relationships: The mediating roles of rumination and relationship satisfaction. *Psychology of Women Quarterly*, *38*(4), 535-550. https://doi.org/10.1177/0361684313517866

Li, J. B., Dou, K., Situ, Q. M., Salcuni, S., Wang, Y. J., & Friese, M. (2019). Anger rumination partly accounts for the association between trait self-control and aggression. *Journal of Research in Personality*, *81*, 207-223. https://doi.org/10.1016/j.jrp.2019.06.011

Li, R., Yang, R., Huang, M., & Xia, L. X. (2022). The longitudinal effect of violent attitude on physical aggression and the underlying motivational mechanisms. *Personality and Individual Differences*, *188*. https://doi.org/10.1016/j.paid.2021.111476

Lilly, M. M., & Mercer, M. C. (2014). The interaction of emotion regulation and world assumptions in predicting female intimate partner violence perpetration. *Partner Abuse*, *5*(4), 439-457. https://doi.org/10.1891/1946-6560.5.4.439

Lin, P. Y., Lin, H. C., Lin, P. C., Yen, J. Y., & Ko, C. H. (2020). The association between Emotional Regulation and Internet Gaming Disorder. *Psychiatry Research*, *289*. https://doi.org/10.1016/j.psychres.2020.113060

Logoz, F., Eggenberger, L., Komlenac, N., Schneeberger, M., Ehlert, U., & Walther, A. (2023). How do traditional masculinity ideologies and emotional competence relate to aggression and physical domestic violence in cisgender men? *Frontiers in Psychology*, *14*. https://doi.org/10.3389/fpsyg.2023.1100114

Long, K., Felton, J. W., Lilienfeld, S. O., & Lejuez, C. W. (2014). The role of emotion regulation in the relations between psychopathy factors and impulsive and premeditated aggression. *Personality Disorders: Theory, Research, and Treatment*, *5*(4), 390-396. https://doi.org/10.1037/per0000085

Mancke, F., Herpertz, S. C., Kleindienst, N., & Bertsch, K. (2017). Emotion dysregulation and trait anger sequentially mediate the association between borderline personality disorder and aggression. *Journal of Personality Disorders*, *31*(2), 256-272. https://doi.org/10.1521/pedi_2016_30_247

Mansfield, A. K., Addis, M. E., Cordova, J. V., & Dowd, L. (2009). Emotional skillfulness as a key mediator of aggression. *Journal of Aggression, Maltreatment and Trauma*, *18*(3), 221-247. https://doi.org/10.1080/10926770902809811

Marín-Morales, A., Pérez-García, M., Catena-Martínez, A., & Verdejo-Román, J. (2022). Emotional regulation in male batterers when faced with pictures of intimate partner violence. Do they have a problem with suppressing or experiencing emotions? *Journal of Interpersonal Violence*, *37*(11), NP10271-NP10295. https://doi.org/10.1177/0886260520985484

Martino, F., Caselli, G., Berardi, D., Fiore, F., Marino, E., Menchetti, M., Prunetti, E., Ruggiero, G. M., Sasdelli, A., Selby, E., Sassaroli, S. (2015). Anger rumination and aggressive behaviour in borderline personality disorder. *Personality and Mental Health*, *9*(4), 277-287. https://doi.org/10.1002/pmh.1310

Martino, F., Caselli, G., Di Tommaso, J., Sassaroli, S., Spada, M. M., Valenti, B., Berardi, D., Sasdelli, A., & Menchetti, M. (2018). Anger and depressive ruminations as predictors of dysregulated behaviours in borderline personality disorder. *Clinical Psychology and Psychotherapy*, *25*(2), 188-194. https://doi.org/10.1002/cpp.2152

Massa, A. A., Eckhardt, C. I., Sprunger, J. G., Parrott, D. J., & Subramani, O. S. (2019). Trauma cognitions and partner aggression: Anger, hostility, and rumination as intervening mechanisms. *Psychology of Violence*, *9*(4), 392-399. https://doi.org/10.1037/vio0000127

Maxwell, J. P., Moores, E., & Chow, C. C. F. (2007). Anger rumination and self-reported aggression amongst British and Hong Kong Chinese athletes: A cross cultural comparison. *International Journal of Sport and Exercise Psychology*, *5*(1), 9-27. https://doi.org/10.1080/1612197X.2008.9671810

Mellat, N., Ebrahimi Ghavam, S., Gholamali Lavasani, M., Moradi, M., & Sadipour, E. (2023). The role of cognitive, emotional, and spiritual development in adult psychological well-being. *Journal of Spirituality in Mental Health*, *25*(1), 31-54. https://doi.org/10.1080/19349637.2022.2121239

Miles, S. R., Menefee, D. S., Wanner, J., Teten Tharp, A., & Kent, T. A. (2016). The relationship between emotion dysregulation and impulsive aggression in veterans with posttraumatic stress disorder symptoms. *Journal of Interpersonal Violence*, *31*(10), 1795-1816. https://doi.org/10.1177/0886260515570746

Oliveros, A. D., & Coleman, A. S. (2021). Does emotion regulation mediate the relation between family-of-origin violence and intimate partner violence? *Journal of Interpersonal Violence*, *36*(19), 9416-9435. https://doi.org/10.1177/0886260519867146

Orozco-Vargas, A. E., Venebra-Muñoz, A., Aguilera-Reyes, U., & García-López, G. I. (2021). The mediating role of emotion regulation strategies in the relationship between family of origin violence and intimate partner violence. *Psicologia: Reflexao e Critica*, *34*(1). https://doi.org/10.1186/s41155-021-00187-8

Ortiz, E., Shorey, R. C., & Cornelius, T. L. (2015). An examination of emotion regulation and alcohol use as risk factors for female-perpetrated dating violence. *Violence and Victims*, *30*(3), 417-431. https://doi.org/10.1891/0886-6708.VV-D-13-00173

Peled, M., & Moretti, M. M. (2010). Ruminating on rumination: Are rumination on anger and sadness differentially related to aggression and depressed mood? *Journal of Psychopathology and Behavioral Assessment*, *32*(1), 108-117. https://doi.org/10.1007/s10862-009-9136-2

Peters, J. R., Smart, L. M., Eisenlohr‐Moul, T. A., Geiger, P. J., Smith, G. T., & Baer, R. A. (2015). Anger rumination as a mediator of the relationship between mindfulness and aggression: The utility of a multidimensional mindfulness model. *Journal of Clinical Psychology*, *71*(9), 871-884. https://doi.org/10.1002/jclp.22189

Pickett, S. M., Parkhill, M. R., Kirwan, M., Aho, K. M., & Nguyen, D. (2017). The Impact of Individual Difference Factors on Men’s Competitive Intent Against a Female Confederate Following Social Stress: A Precursor for Aggression. *Journal of Men's Studies*, *25*(1), 25-43. https://doi.org/10.1177/1060826516636085

Pollard, D. L., & Cantos, A. L. (2021). Attachment, emotion dysregulation and physical ipv in predominantly hispanic, young adult couples. *International Journal of Environmental Research and Public Health*, *18*(14). https://doi.org/10.3390/ijerph18147241

Preston, O. C., & Anestis, J. C. (2020). The indirect relationships between psychopathic traits and proactive and reactive aggression through empathy and emotion dysregulation. *Journal of Psychopathology and Behavioral Assessment*, *42*(3), 409-423. https://doi.org/10.1007/s10862-019-09760-z

Price, R. K., Bell, K. M., & Lilly, M. (2014). The interactive effects of PTSD, emotion regulation, and anger management strategies on female-perpetrated IPV. *Violence and Victims*, *29*(6), 907-926. https://doi.org/10.1891/0886-6708.VV-D-12-00123

Pugliese, C. E., Fritz, M. S., & White, S. W. (2015). The role of anger rumination and autism spectrum disorder–linked perseveration in the experience of aggression in the general population. *Autism*, *19*(6), 704-712. https://doi.org/10.1177/1362361314548731

Puhalla, A., er, A., & McCloskey, M. S. (2020). The relationship between physiological reactivity to provocation and emotion dysregulation with proactive and reactive aggression. *Biological Psychology*, *155*. https://doi.org/10.1016/j.biopsycho.2020.107931

Quan, F., Wang, L., Gong, X., Lei, X., Liang, B., & Zhang, S. (2022). Hostile Attribution Bias and Anger Rumination Sequentially Mediate the Association Between Trait Anger and Reactive Aggression. *Frontiers in Psychology*, *12*. https://doi.org/10.3389/fpsyg.2021.778695

Quan, F., Yang, R., & Xia, L. X. (2021). The longitudinal relationships among agreeableness, anger rumination, and aggression. *Current Psychology*, *40*(1), 9-20. https://doi.org/10.1007/s12144-020-01030-6

Quan, F., Yang, R., Zhu, W., Wang, Y., Gong, X., Chen, Y., Dong, Y., & Xia, L.-X. (2019). The relationship between hostile attribution bias and aggression and the mediating effect of anger rumination. *Personality and Individual Differences*, *139*, 228-234. https://doi.org/10.1016/j.paid.2018.11.029

Ray, T. N., & Parkhill, M. R. (2020). Examining disgust and emotion regulation difficulties as components of aggression toward perceived gay men. *Psychology of Violence*, *10*(4), 462-471. https://doi.org/10.1037/vio0000265

Rogier, G., Garofalo, C., & Velotti, P. (2019). Is Emotional Suppression Always Bad? A Matter of Flexibility and Gender Differences. *Current Psychology*, *38*(2), 411-420. https://doi.org/10.1007/s12144-017-9623-7

Sanchez-Ruiz, M. J., & Baaklini, A. (2018). Individual and social correlates of aggressive behavior in Lebanese undergraduates: The role of trait emotional intelligence. *J Soc Psychol*, *158*(3), 350-360. https://doi.org/10.1080/00224545.2017.1353476

Sarrate-Costa, C., Lila, M., Comes-Fayos, J., Moya-Albiol, L., & Romero-Martínez, Á. (2023). Reduced vagal tone in intimate partner violence perpetrators is partly explained by anger rumination. *Current Psychology*, *42*(33), 29603-29615. https://doi.org/10.1007/s12144-022-03994-z

Scott, L. N., Stepp, S. D., & Pilkonis, P. A. (2014). Prospective associations between features of borderline personality disorder, emotion dysregulation, and aggression. *Personality Disorders: Theory, Research, and Treatment*, *5*(3), 278-288. https://doi.org/10.1037/per0000070

Shamsipour, H., Bazani, M., Tashkeh, M., & Mohammadi, S. (2018). The role of negative affects and emotion dysregulation in aggression. *Journal of Practice in Clinical Psychology*, *6*(4), 249-256. https://doi.org/10.32598/jpcp.6.4.249

Shorey, R., McNulty, J., Moore, T., Stuart, G., Shorey, R. C., McNulty, J. K., Moore, T. M., & Stuart, G. L. (2015). Emotion Regulation Moderates the Association Between Proximal Negative Affect and Intimate Partner Violence Perpetration. *Prevention Science*, *16*(6), 873-880. https://doi.org/10.1007/s11121-015-0568-5

Shorey, R. C., Brasfield, H., Febres, J., & Stuart, G. L. (2011a). An examination of the association between difficulties with emotion regulation and dating violence perpetration. *Journal of Aggression, Maltreatment & Trauma*, *20*(8), 870-885. https://doi.org/10.1080/10926771.2011.629342

Shorey, R. C., Cornelius, T. L., & Idema, C. (2011b). Trait anger as a mediator of difficulties with emotion regulation and female-perpetrated psychological aggression. *Violence and Victims*, *26*(3), 271-282. https://doi.org/10.1891/0886-6708.26.3.271

Shorey, R. C., Elmquist, J., Zucosky, H., Febres, J., Brasfield, H., & Stuart, G. L. (2014). Experiential avoidance and male dating violence perpetration: An initial investigation. *Journal of Contextual Behavioral Science*, *3*(2), 117-123. https://doi.org/10.1016/j.jcbs.2014.02.003

Sotelo, J., & Babcock, J. (2013). BIS/BAS Variables as Moderators of the Rumination-Intimate Partner Violence Link. *Journal of Family Violence*, *28*(3), 233-242. https://doi.org/10.1007/s10896-013-9500-6

Stappenbeck, C. A., Davis, K. C., Cherf, N., Gulati, N. K., & Kajumulo, K. F. (2016). Emotion regulation difficulties moderate the association between heavy episodic drinking and dating violence perpetration among college men. *Journal of Aggression, Maltreatment & Trauma*, *25*(9), 921-935. https://doi.org/10.1080/10926771.2016.1232328

Terzi, L., Martino, F., Berardi, D., Bortolotti, B., Sasdelli, A., & Menchetti, M. (2017). Aggressive behavior and self-harm in Borderline Personality Disorder: The role of impulsivity and emotion dysregulation in a sample of outpatients. *Psychiatry Research*, *249*, 321-326. https://doi.org/10.1016/j.psychres.2017.01.011

Thiessen, M. S., Walsh, Z., Bird, B. M., & Lafrance, A. (2018). Psychedelic use and intimate partner violence: The role of emotion regulation. *Journal of Psychopharmacology*, *32*(7), 749-755. https://doi.org/10.1177/0269881118771782

Trombetta, T., Balocco, V., Santoniccolo, F., Paradiso, M. N., & Rollè, L. (2023). Internalized Homonegativity, Emotion Dysregulation, and Isolating Behaviors Perpetration among Gay and Lesbian Couples. *International Journal of Environmental Research and Public Health*, *20*(2). https://doi.org/10.3390/ijerph20021593

Tull, M. T., Jakupcak, M., Paulson, A., & Gratz, K. L. (2007). The role of emotional inexpressivity and experiential avoidance in the relationship between posttraumatic stress disorder symptom severity and aggressive behavior among men exposed to interpersonal violence. *Anxiety, Stress and Coping*, *20*(4), 337-351. https://doi.org/10.1080/10615800701379249

Turner, K. A., & White, B. A. (2015). Contingent on contingencies: Connections between anger rumination, self-esteem, and aggression. *Personality and Individual Differences*, *82*, 199-202. https://doi.org/10.1016/j.paid.2015.03.023

Velotti, P., Casselman, R. B., Garofalo, C., & McKenzie, M. D. (2017). Unique Associations Among Emotion Dysregulation Dimensions and Aggressive Tendencies: A Multisite Study. *Violence & Victims*, *32*(5), 791-810. https://doi.org/10.1891/0886-6708.VV-D-16-00079

Velotti, P., Garofalo, C., Petrocchi, C., Cavallo, F., Popolo, R., & Dimaggio, G. (2016). Alexithymia, emotion dysregulation, impulsivity and aggression: A multiple mediation model. *Psychiatry Research*, *237*, 296-303. https://doi.org/10.1016/j.psychres.2016.01.025

Velotti, P., Rogier, G., & Sarlo, A. (2020). Pathological narcissism and aggression: The mediating effect of difficulties in the regulation of negative emotions. *Personality and Individual Differences*, *155*. https://doi.org/10.1016/j.paid.2019.109757

Wahlstrom, L. C., Scott, J. P., Tuliao, A. P., DiLillo, D., & McChargue, D. E. (2015). Posttraumatic stress disorder symptoms, emotion dysregulation, and aggressive behavior among incarcerated methamphetamine users. *Journal of Dual Diagnosis*, *11*(2), 118-127. https://doi.org/10.1080/15504263.2015.1025026

Wang, X., Yang, L., Yang, J., Gao, L., Zhao, F., Xie, X., & Lei, L. (2018). Trait anger and aggression: A moderated mediation model of anger rumination and moral disengagement. *Personality and Individual Differences*, *125*, 44-49. https://doi.org/10.1016/j.paid.2017.12.029

Wang, Y., Cao, S., Zhang, Q., & Xia, L. X. (2020). The longitudinal relationship between angry rumination and reactive–proactive aggression and the moderation effect of consideration of future consequences‐immediate. *Aggressive Behavior*, *46*(6), 476-488. https://doi.org/10.1002/ab.21913

Watkins, L. E., Schumacher, J. A., & Coffey, S. F. (2016). A preliminary investigation of the relationship between emotion dysregulation and partner violence perpetration among individuals with PTSD and alcohol dependence. *Journal of Aggression, Maltreatment & Trauma*, *25*(3), 305-314. https://doi.org/10.1080/10926771.2015.1129657

Wei, D., Hou, F., Cao, W., Hao, C., Gu, J., Peng, L., & Li, J. (2020). Effects of emotion regulation and perpetrator-victim roles in intimate partner violence on mental health problems among men who have sex with men in China. *Epidemiology and Psychiatric Sciences*, *29*. https://doi.org/10.1017/S2045796020000712

White, B. A., & Turner, K. A. (2014). Anger rumination and effortful control: Mediation effects on reactive but not proactive aggression. *Personality and Individual Differences*, *56*, 186-189. https://doi.org/10.1016/j.paid.2013.08.012

Yang, Y. (2020). Aggression and somatic symptoms: The role of emotion regulation and distress tolerance. *International Journal of Behavioral Medicine*, *27*(4), 466-474. https://doi.org/10.1007/s12529-020-09885-6

**References for Table S2: Emotion Regulation Measures**

Bond, F. W., Hayes, S. C., Baer, R. A., Carpenter, K. M., Guenole, N., Orcutt, H. K., Waltz, T., & Zettle, R. D. (2011). Preliminary psychometric properties of the Acceptance and Action Questionnaire–II: A revised measure of psychological inflexibility and experiential avoidance. *Behavior therapy*, *42*(4), 676-688. https://doi.org/10.1016/j.beth.2011.03.007

Briere, J. (2000). *Inventory of Altered Self-Capacities professional manual*. PAR.

Brinker, J. K., & Dozois, D. J. (2009). Ruminative thought style and depressed mood. *Journal of Clinical Psychology*, *65*(1), 1-19. https://doi.org/10.1002/jclp.20542

Caprara, G. V. (1986). Indicators of aggression: The dissipation-rumination scale. *Personality and Individual Differences*, *7*(6), 763-769. https://doi.org/10.1016/0191-8869(86)90074-7

Catanzaro, S. J., & Mearns, J. (1990). Measuring generalized expectancies for negative mood regulation: Initial scale development and implications. *Journal of Personality Assessment*, *54*(3-4), 546-563. https://doi.org/10.1080/00223891.1990.9674019

Denson, T. F., Pedersen, W. C., & Miller, N. (2006). The displaced aggression questionnaire. *Journal of Personality and Social Psychology, 90*(6), 1032–1051. https://doi.org/10.1037/0022-3514.90.6.1032

Garnefski, N., Kraaij, V., & Spinhoven, P. (2001). Negative life events, cognitive emotion regulation and emotional problems. *Personality and Individual Differences*, *30*(8), 1311-1327. https://doi.org/10.1016/S0191-8869(00)00113-6

Gratz, K. L., & Roemer, L. (2004). Multidimensional assessment of emotion regulation and dysregulation: Development, factor structure, and initial validation of the difficulties in emotion regulation scale. *Journal of Psychopathology and Behavioral Assessment*, *26*, 41-54. https://doi.org/10.1023/B:JOBA.0000007455.08539.94

Gross, J. J., & John, O. P. (2003). Individual differences in two emotion regulation processes: implications for affect, relationships, and well-being. *Journal of Personality and Social Psychology*, *85*(2), 348. https://doi.org/10.1037/0022-3514.85.2.348

Hayes, S. C., Strosahl, K., Wilson, K. G., Bissett, R. T., Pistorello, J., Toarmino, D., ... & McCurry, S. M. (2004). Measuring experiential avoidance: A preliminary test of a working model. *The Psychological Record*, *54*, 553-578. <https://doi.org/10.1007/BF03395492>

Hofmann, S. G., & Kashdan, T. B. (2010). The affective style questionnaire: development and psychometric properties. *Journal of Psychopathology and Behavioral Assessment*, *32*, 255-263. https://doi.org/10.1007/s10862-009-9142-4

Larsen, R. J., & Prizmic, Z. (2004). Affect regulation. In R. Baumeister & K. Vohs (Eds.), *Handbook of self-regulation research* (pp.40–60). Nueva York: Guilford.

Mezzich, A. C., Tarter, R. E., Giancola, P. R., & Kirisci, L. (2001). The dysregulation inventory: A new scale to assess the risk for substance use disorder. *Journal of Child & Adolescent Substance Abuse*, *10*(4), 35-43. <https://doi.org/10.1300/J029v10n04_04>

Nolen-Hoeksema, S., & Morrow, J. (1991). A prospective study of depression and posttraumatic stress symptoms after a natural disaster: the 1989 Loma Prieta Earthquake. *Journal of Personality and Social Psychology*, *61*(1), 115. https://doi.org/10.1037/0022-3514.61.1.115

Peled, M., & Moretti, M. M. (2007). Rumination on anger and sadness in adolescence: Fueling of fury and deepening of despair. *Journal of Clinical Child and Adolescent Psychology*, *36*(1), 66-75. https://doi.org/10.1080/15374410709336569

Salovey, P., Mayer, J. D., Goldman, S. L., Turvey, C., & Palfai, T. P. (1995). Emotional attention, clarity, and repair: Exploring emotional intelligence using the Trait Meta-Mood Scale. In J. W. Pennebaker (Ed.), *Emotion, disclosure, & health* (pp. 125–154). American Psychological Association. https://doi.org/10.1037/10182-006

Sukhodolsky, D. G., Golub, A., & Cromwell, E. N. (2001). Development and validation of the anger rumination scale. *Personality and Individual Differences*, *31*(5), 689-700. https://doi.org/10.1016/S0191-8869(00)00171-9

Trapnell, P. D., & Campbell, J. D. (1999). Private self-consciousness and the five-factor model of personality: distinguishing rumination from reflection. *Journal of Personality and Social Psychology*, *76*(2), 284. https://doi.org/10.1037/0022-3514.76.2.284

Treynor, W., Gonzalez, R., & Nolen-Hoeksema, S. (2003). Rumination reconsidered: A psychometric analysis. *Cognitive Therapy and Research*, *27*, 247-259. <https://doi.org/10.1023/A:1023910315561>

Watson, M., & Greer, S. (1983). Development of a questionnaire measure of emotional control. *Journal of Psychosomatic Research, 27*(4), 299-305. https://doi.org/10.1016/0022-3999(83)90052-1

**References for Table S3: Aggression Measures**

Achenbach, T. M., & Rescorla, L. (2003). *Manual for the ASEBA adult forms & profiles.* University of Vermont, Research Center for Children, Youth, & Families.

Achenbach, T. M., & Rescorla, L. A. (2001). Manual for ASEBA school-age forms and profiles. *University of Vermont, Research Centre for Children, Youth, & Families*.

Borders, A., Barnwell, S. S., & Earleywine, M. (2007). Alcohol‐aggression expectancies and dispositional rumination moderate the effect of alcohol consumption on alcohol‐related aggression and hostility. *Aggressive Behavior: Official Journal of the International Society for Research on Aggression*, *33*(4), 327-338. https://doi.org/ https://doi.org/10.1002/ab.20187

Borjesson, W. I., Aarons, G. A., & Dunn, M. E. (2003). Development and confirmatory factor analysis of the abuse within intimate relationships scale. *Journal of Interpersonal Violence*, *18*(3), 295-309. https://doi.org/10.1177/0886260502250089

Boxer, P., Tisak, M. S., & Goldstein, S. E. (2004). Is it bad to be good? An exploration of aggressive and prosocial behavior subtypes in adolescence. *Journal of Youth and Adolescence*, *33*, 91-100. https://doi.org/10.1023/B:JOYO.0000013421.02015.ef

Bryant, F. B., & Smith, B. D. (2001). Refining the architecture of aggression: A measurement model for the Buss–Perry Aggression Questionnaire. *Journal of Research in Personality*, *35*(2), 138-167. https://doi.org/10.1006/jrpe.2000.2302

Buss, A. H., & Durkee, A. (1957). An inventory for assessing different kinds of hostility. *Journal of Consulting Psychology, 21*(4), 343–349. https://doi.org/10.1037/h0046900

Buss, A. H., & Perry, M. (1992). The aggression questionnaire. *Journal of Personality and Social Psychology*, *63*(3), 452. https://doi.org/10.1037/0022-3514.63.3.452

Buss, A. H., & Warren, W. L. (2000). *The aggression questionnaire manual*. Western Psychological Services.

Caprara, G. V. (1986). Indicators of aggression: The dissipation–rumination scale. *Personality and Individual Differences, 7,* 763–769. <https://doi.org/10.1016/0191-8869(86)90074-7>

Caprara, G. V., Mazzotti, E., & Prezza, M. (1990). Una scala per la misura dell'atteggiamento verso la violenza [A scale for measuring attitude toward violence]. *Giornale Italiano di Psicologia, 17*(3), 107-120. http://www.mulino.it/edizioni/riviste/issn/0390-5349

Coccaro, E. F., Berman, M. E., & Kavoussi, R. J. (1997). Assessment of life history of aggression: development and psychometric characteristics. *Psychiatry Research*, *73*(3), 147-157. https://doi.org/10.1016/S0165-1781(97)00119-4

Dyar, C., Messinger, A. M., Newcomb, M. E., Byck, G. R., Dunlap, P., & Whitton, S. W. (2021). Development and initial validation of three culturally sensitive measures of intimate partner violence for sexual and gender minority populations. *Journal of Interpersonal Violence, 36*(15–16), NP8824–NP8851. <https://doi.org/10.1177%2F0886260519846856>

Foshee, V. A., Bauman, K. E., Arriaga, X. B., Helms, R. W., Koch, G. G., & Linder, G. F. (1998). An evaluation of Safe Dates, an adolescent dating violence prevention program. *American Journal of Public Health*, *88*(1), 45-50. https://doi.org/10.2105/AJPH.88.1.45

Gillikin, C., Habib, L., Evces, M., Bradley, B., Ressler, K. J., & Sanders, J. (2016). Trauma exposure and PTSD symptoms associate with violence in inner city civilians. *Journal of Psychiatric Research*, *83*, 1-7. <https://doi.org/10.1016/J.JPSYCHIRES.2016.07.027>

Isobe, M., & Hishinuma, Y. (2007). Overt and relational aggression and impression formation in Japanese university students. *Japanese Journal of Personality*, *15*(3), 290-300.

Koss, M. P., Abbey, A., Campbell, R., Cook, S., Norris, J., Testa, M., Ullman, S., West, C., & White, J. (2007). Revising the SES: A collaborative process to improve assessment of sexual aggression and victimization. *Psychology of Women Quarterly*, *31*(4), 357-370. https://doi.org/10.1111/j.1471-6402.2007.00385.x

Koss, M. P., Gidycz, C. A., & Wisniewski, N. (1987). The scope of rape: Incidence and prevalence of sexual aggression and victimization in a national sample of higher education students. *Journal of Consulting and Clinical Psychology*, *55*(2), 162. https://doi.org/10.1037/0022-006X.55.2.162

Lin, T. K., Weng, C.-Y., Wang, W.-C., Chen, C.-C., Lin, I.-M., & Lin, C.-L. (2008). Hostility trait and vascular dilatory functions in healthy Taiwanese. *Journal of Behavioral Medicine*, *31*, 517-524. https://doi.org/10.1007/s10865-008-9177-0

Little, T. D., Henrich, C. C., Jones, S. M., & Hawley, P. H. (2003). Disentangling the “whys” from the “whats” of aggressive behaviour. *International Journal of Behavioral Development*, *27*(2), 122-133. https://doi.org/10.1080/01650250244000128

Maldonado, A. I., Farzan-Kashani, J., Sun, S., Pitts, S. C., Lorenzo, J. M., Barry, R. A., & Murphy*, C. M. (2022). Psychometric properties and factor analysis of a short form of the multidimensional measure of emotional abuse. *Journal of Interpersonal Violence*, *37*(7-8), NP4905-NP4930. https://doi.org/10.1177/0886260520957668

Marsee, M. A., & Frick, P. J. (2007). Exploring the cognitive and emotional correlates to proactive and reactive aggression in a sample of detained girls. *Journal of Abnormal Child Psychology, 35*(6), 969–981. <https://doi.org/10.1007/S20802-007-9147-y>

Marsee, M. A., Barry, C. T., Childs, K. K., Frick, P. J., Kimonis, E. R., Muñoz, L. C., Aucoin, K. J., Fassnacht, G. M., Kunimatsu, M. M., & Lau, K. S. L. (2011). Assessing the forms and functions of aggression using self-report: Factor structure and invariance of the Peer Conflict Scale in youths. *Psychological Assessment, 23(*3), 792–804. <https://doi.org/10.1037/a0023369>

Monahan, J., & Steadman, H. J. (1994). *Violence and mental disorder: Developments* *in risk assessment.* University of Chicago Press.

Morales, J. R., & Crick, N. R. (1998). *Self-report measure of aggression and victimization* [Unpublished manuscript]. University of Minnesota. https://doi.org/10.1037/t29632-000.

Murray, A. L., Eisner, M., Ribeaud, D., & Booth, T. (2022). Validation of a brief measure of aggression for ecological momentary assessment research: The aggression-ES-A. *Assessment*, *29*(2), 296-308. https://doi.org/10.1177/107319112097685

Parkhill, M. R., & Abbey, A. (2008). Does alcohol contribute to the confluence model of sexual assault perpetration? *Journal of Social and Clinical Psychology*, *27*(6), 529-554. https://doi.org/10.1521/jscp.2008.27.6.529

Parrott, D. J., & Peterson, J. L. (2008). What motivates hate crimes based on sexual orientation? Mediating effects of anger on antigay aggression. *Aggressive Behavior: Official Journal of the International Society for Research on Aggression*, *34*(3), 306-318. http://dx.doi.org/10.1002/ab.20239

Raine, A., Dodge, K., Loeber, R., Gatzke‐Kopp, L., Lynam, D., Reynolds, C., Stouthamer‐Loeber, M., & Liu, J. (2006). The reactive–proactive aggression questionnaire: Differential correlates of reactive and proactive aggression in adolescent boys. *Aggressive Behavior: Official Journal of the International Society for Research on Aggression*, *32*(2), 159-171. https://doi.org/10.1002/ab.20115

Roderick, T., McCammon, S. L., Long, T. E., & Allred, L. J. (1998). Behavioral aspects of homonegativity. *Journal of Homosexuality*, *36*(1), 79-88. http://dx.doi.org/10.1300/J082v36n01_05

Sleath, E., Walker, K., & Tramontano, C. (2018). Factor structure and validation of the controlling behaviors scale–revised and revised conflict tactics scale. *Journal of Family Issues*, *39*(7), 1880-1903. https://doi.org/10.1177/0192513X1772972

Stanford, M. S., Houston, R. J., Mathias, C. W., Villemarette-Pittman, N. R., Helfritz, L. E., & Conklin, S. M. (2003). Characterizing aggressive behavior. *Assessment*, *10*(2), 183-190. https://doi.org/10.1177/1073191103010002009

Stephenson, R., & Finneran, C. (2013). The IPV-GBM scale: A new scale to measure intimate partner violence among gay and bisexual men. *PloS one*, *8*(6), e62592. https://doi.org/https://doi.org/10.1371/journal.pone.0062592

Straus, M. A., & Douglas, E. M. (2004). A short form of the Revised Conflict Tactics Scales, and typologies for severity and mutuality. *Violence and Victims, 19*(5), 507-520. 10.1891/vivi.19.5.507.63686

Straus, M. A., Hamby, S. L., Boney-McCoy, S., & Sugarman, D. B. (1996). The revised conflict tactics scales (CTS2) development and preliminary psychometric data. *Journal of Family Issues*, *17*(3), 283-316. https://doi.org/10.1177/01925139601700300

Swahn, M. H., Simon, T. R., Arias, I., & Bossarte, R. M. (2008). Measuring sex differences in violence victimization and perpetration within date and same-sex peer relationships. *Journal of Interpersonal Violence*, *23*(8), 1120-1138. https://doi.org/https://doi.org/10.1177/0886260508314086

Valdez-Santiago, R., Híjar-Medina, M. C., Salgado de Snyder, V. N., Rivera-Rivera, L., Avila-Burgos, L., & Rojas, R. (2006). Escala de violencia e índice de severidad: una propuesta metodológica para medir la violencia de pareja en mujeres mexicanas. *Salud Pública de México*, *48*, s221-s231. https://doi.org/10.1590/S0036-36342006000800002

Whitmire, L., Harlow, L., Quina, K., & Morokoff, P. (1999). *Childhood trauma and HIV: Women at risk.* Brunner/Mazel Inc.

Zahedifar, S., Najarian, B., & Shokrkon, H. (2000). Construction and validation of a scale for the measurement of aggression. *Journal of Educational Sciences, 7*(1), 73-102. doi: 10.22055/edus.2000.16084
